# Multidimensional molecular profiling reveals decidual and circulating NK cell dynamics in early and late pregnancy

**DOI:** 10.1016/j.ebiom.2026.106495

**Published:** 2026-10-01

**Authors:** Jennifer R. Habel, Thi H.O. Nguyen, Lilith F. Allen, Morgan J. Skinner, Lukasz Kedzierski, E Kaitlynn Allen, Shihan Li, Natasha de Alwis, Ruth R. Hagen, Karen Doggett, Xiaoxiao Jia, Ilariya Tarasova, Anastasia A. Minervina, Mikhail V. Pogorelyy, Ashleigh I. Holloway, Nikita L. Mathew, Philippa M. Saunders, Allison Clatch, Maximilien Evrard, Calvin Xu, Hui-Fern Koay, Md Abdullah-Al-Kamran Khan, Laura K. Mackay, Alexander D. Barrow, Celia Douros, Theo Karapanagiotidis, Suellen Nicholson, Katherine Bond, Deborah A. Williamson, Lisa Hui, Martha Lappas, Susan Walker, Sandra E. Nicholson, Natalie J. Hannan, Andrew G. Brooks, Jan Schroeder, Jeremy Chase Crawford, Paul G. Thomas, Louise C. Rowntree, Katherine Kedzierska

**Affiliations:** a Department of Microbiology and Immunology, University of Melbourne at the Peter Doherty Institute for Infection and Immunity, Melbourne, Victoria, Australia; b Department of Immunology, St. Jude Children’s Research Hospital, Memphis, TN, USA; c Computational Sciences Initiative, Department of Microbiology and Immunology, University of Melbourne at the Peter Doherty Institute for Infection and Immunity, Melbourne, Victoria, Australia; d Department of Obstetrics, Gynaecology and Newborn Health, University of Melbourne, Heidelberg, Victoria, Australia; e Mercy Perinatal, Mercy Hospital for Women, Heidelberg, Victoria, Australia; f The Walter and Eliza Hall Institute of Medical Research, Melbourne, Victoria, Australia; g Department of Medical Biology, University of Melbourne, Melbourne, Victoria, Australia; h Immunology and Vaccine Development Program, Vaccine and Infectious Disease Division, Fred Hutchinson Cancer Center, Seattle, WA, USA; i Victorian Infectious Diseases Reference Laboratory at the Peter Doherty Institute for Infection and Immunity, Melbourne, Victoria, Australia; j Department of Microbiology, Royal Melbourne Hospital, Melbourne, Victoria, Australia; k Victorian Infectious Diseases Service, Royal Melbourne Hospital at the Peter Doherty Institute for Infection and Immunity, Melbourne, Victoria, Australia; l Department of Infectious Diseases, University of Melbourne at the Peter Doherty Institute for Infection and Immunity, Melbourne, Victoria, Australia; m School of Medicine, University of St Andrews, St Andrews, SCT, UK; n Northern Health, Epping, Victoria, Australia; o Reproductive Epidemiology Group, Murdoch Children’s Research Institute, Parkville, Victoria, Australia; p Department of Host-Microbe Interactions, St. Jude Children’s Research Hospital, Memphis, TN, USA

**Keywords:** NK cells, Pregnancy, Decidua, Cytomegalovirus, Multiomics

## Abstract

**Background:**

Human pregnancy presents a unique physiological state that allows for growth of an antigenically dissimilar foetus. The immune system plays a key role in establishing and maintaining successful pregnancy, yet detailed understanding of immunological responses in pregnancy is lacking.

**Methods:**

We defined the immunological landscape in pregnancy by screening >350 markers on NK cells, CD4^+^, CD8^+^ and γδ T cells, B cells and monocytes in third-trimester pregnancy peripheral blood, cord blood and decidua, and by performing single-cell RNA sequencing (scRNAseq), including 130 CITE-seq antibodies, on NK cells from the blood and decidua of third-trimester pregnancies. We validated our findings in additional blood and decidua samples from first- and third-trimester pregnancies. Blood samples from non-pregnant women were used as a control group for comparison.

**Findings:**

Our multiomic approaches revealed altered surface proteomes in lymphoid and myeloid immune subsets during third-trimester pregnancy, in comparison to non-pregnant women. In the blood during pregnancy, circulating NK cells displayed increased *SOCS1* (FDR < 0.001) and *AREG* (FDR < 0.001), both involved in tightly regulating immune responses, as well as *HLA-D* family (FDR < 0.05–0.0001) gene expression, with increased CCR5 (p < 0.01–0.001) and HLA-DR (p < 0.01–0.001), and decreased CD9 (p < 0.001), CD36 (p < 0.05–0.001), and CX3CR1 (p < 0.05) at the protein level, validated in samples from first- and third-trimester pregnancies. Our analysis of decidual NK (dNK) cells demonstrated a decline in the dNK1 subset between first-trimester decidua and third-trimester decidua basalis (p < 0.0001) and parietalis (p < 0.01), and identified an adaptive-like subset with high expression of cytotoxic molecules, which could be distinguished from the previously described dNK1-3 subsets and was enriched in decidua from women with a history of cytomegalovirus infection (p < 0.05). Functionally, we observed enhanced control of IFN-γ secretion in response to proinflammatory IL-12/IL-18 cytokines in pregnancy (p < 0.05–0.01), however induction of SOCS1 protein expression remained similar between pregnant and non-pregnant women.

**Interpretation:**

Overall, our study provides key insights into immunity in pregnancy and reveals fundamental differences in the NK cell compartment. Understanding pregnancy-specific immunology fills important knowledge gaps to better inform the rational design of vaccines, immunotherapies and identification of biomarkers for safe pregnancy outcomes.

**Funding:**

Australian National Health and Medical Research Council, Australian Medical Research Future Fund, and the National Institute of Allergy and Infectious Diseases.

**Research in context**

**Evidence before this study**

During pregnancy the immune system must perform a balancing act, whereby immune activation is required in both early and late pregnancy to develop the maternal–foetal interface and prepare for foetal expulsion (i.e., birth), however tight immune regulation is required to prevent detrimental pregnancy outcomes or complications. Adding to the complexity, maternal immunity needs to maintain optimal functionality to protect from infectious diseases.

Natural killer (NK) cells have key roles in both protection from viral infections and at the maternal–foetal interface. A seminal study defined three decidual NK (dNK) cell subsets in first-trimester pregnancy, and prior to that, the literature suggested an acquired increase in “adaptive-like”, or terminally differentiated, NK cells in the blood and decidua with pregnancy. However, there has been no link between adaptive-like NK cell phenotypes and the three dNK subsets. It is unclear whether the existing three dNK subsets exhibit adaptive-like features, and there is little understanding of how these subsets change between early and late pregnancy.

Additionally, many immunology studies focused on pregnancy analyse the maternal–foetal interface, as it presents an interesting immunological setting. However, outside of pregnancy most immunology studies in humans analyse peripheral blood as a proxy to understanding immune responses more systemically. The lack of pregnancy-focused studies analysing features of immunity in the peripheral blood creates a knowledge gap on the effect pregnancy has on basal immune cell phenotypes that we aim to address in our study.

**Added value of this study**

Using high-dimensional analyses, we reveal a fourth dNK subset with tissue-residency and adaptive-like features that can be distinguished from the three dNK subsets reported previously. dNK4 cells were enriched in decidua from women with a history of cytomegalovirus (CMV) and had altered expression of adhesion and exhaustion markers. We also identified changes to expression of activation and migration markers on NK cells in the peripheral blood during early and late pregnancy. This provides further insight into how pregnancy impacts immune cell phenotypes, and that pregnancy status may be a key consideration when defining immune activation.

**Implications of all the available evidence**

Having identified a unique subset of adaptive-like dNK cells, future studies can further understand their functional relevance, given adaptive-like NK cells in the blood have been linked with cytomegalovirus infections – which are a leading cause of birth defects if infected during pregnancy. Additionally, understanding the baseline immune signatures in pregnancy can inform future analyses that decipher the role of the immune system in complications or infections during pregnancy.

## Introduction

Human pregnancy presents a unique physiological state that allows for growth of an antigenically dissimilar foetus. While there is a partial physical separation of the foetus by amniotic and chorionic membranes, successful human pregnancy requires a tightly regulated immune system to remain non-reactive toward foetal antigens. To achieve this there is a characteristic bias towards type II or tolerogenic immunity during pregnancy (reviewed in ^1^).

Despite the required regulation of immunity, there are key features of immune activation observed in healthy pregnancies. For example, higher proportions of activated HLA-DR^+^CD38^+^ CD8^+^ T cells are reported in maternal peripheral blood.^2^ Within the decidua, CD8^+^ T cells have increased expression of granulysin, while subsets of effector/effector-memory CD8^+^ T cells have lower expression of granzyme B and perforin, as compared to that of pregnancy-matched peripheral blood.^3^ Maternal regulatory CD4^+^ T cells have a vital role in maintaining tolerance of the foetus.^4^ Furthermore, our group recently reported perturbations to the pro-inflammatory interleukin-6 (IL-6) axis on CD4^+^ T cells in pregnancy, which could have implications in pregnancy complications or infectious disease severity.^5^

Pregnancy also causes an increase in regulatory B cells, defined by co-expression of CD24 and CD27 and production of IL-10,^6^ and by the third trimester of pregnancy, there is a substantial reduction in circulating B cells.^7^^,^^8^ Pregnancy mouse models demonstrated reduced numbers of B cell precursors in the bone marrow, and that B cell precursors are less sensitive to IL-7 stimulation, required for their differentiation.^9^^,^^10^ Further to this, oral administration of oestrogen and progesterone for 2 weeks caused reduced B cell lymphopoiesis in mice, suggesting these hormones negatively regulate B cell differentiation.^11^

Innate immune cells are also known to play a role in successful pregnancy. Unconventional T cells make up a minor proportion of circulating T lymphocytes, however successful pregnancy is still linked to their optimal function. Several reports have associated repeated pregnancy loss (RPL) or implantation failure with certain γδ T cell phenotypes, including high CD107a expression,^12^ higher proportions of cytotoxic Vγ9Vδ2 T cells^13^, ^14^, ^15^ and higher frequencies of NKG2D^+^ γδ T cells.^16^ Conversely, higher frequencies of Vδ1 T cells are linked to maternal tolerance and successful pregnancy.^13^^,^^17^ In healthy pregnancies, one report identified an expansion of CD56^+^ γδ T cells in the second and third trimesters, although the significance of this is yet to be described.^18^ Additionally, in early pregnancy, γδ T cells are an important source of the regulatory cytokines IL-10 and TGF-β in the decidua, allowing for optimal invasion of the uterus.^19^^,^^20^

Of all the immune cells, natural killer (NK) cells have the most well-established role in placentation and establishment of foetal vascular supply, which is, in part, regulated by killer cell immunoglobulin-like receptors (KIRs) interacting with HLA-C on foetal-derived trophoblast cells.^21^ Decidual NK cells secrete IFN-γ in response to invading trophoblasts, causing widening of spiral arteries which is essential for optimal blood flow and nutrient exchange in healthy pregnancies.^22^^,^^23^ Consistent with this, genetic variation impacting KIR/HLA-C interactions has been associated with the life-threatening hypertensive disorder pre-eclampsia, often leading to major organ and cardiovascular injury.^24^ At the maternal–foetal interface, three subsets of decidual NK cells have been described in first-trimester pregnancy, which have varying levels of KIR and cytotoxic molecule expression.^25^ In addition to the maternal–foetal interface, maternal peripheral NK cells undergo phenotypic changes, which are, at least in part, caused by signalling from trophoblasts.^26^ Additionally, in first-trimester pregnancies (6–12 weeks), there is an increased frequency of IL-10^+^ CD56^bright^ NK cells in the blood compared to non-pregnant women.^27^ The importance of balanced NK cell activation during pregnancy is exemplified in women with recurrent pregnancy loss, who have increased NK cell cytolytic activity compared to women with no pregnancy loss, suggesting that NK cell cytotoxic functions need to be restrained for healthy and full-term pregnancy.^28^

Despite clear evidence that NK cells play an important role in pregnancy, fundamental knowledge is lacking, including a mechanistic understanding of their altered phenotypes and functions, especially within peripheral blood. Furthermore, an in-depth understanding of broad immunological responses in pregnancy is far from clear, but is key for the rational design of vaccines, immunotherapies and biomarkers for safe pregnancy outcomes. This is particularly relevant given that pregnancy is often an exclusion criterion for clinical trials whereby medications and vaccines can have unknown safety and efficacy in pregnancy. Therefore, to provide in-depth understanding of the immunological landscape of pregnancy, with a focus on NK cells, we used high-dimensional approaches to identify key differentially expressed molecules and investigated their potential roles in altered immune functionality.

## Methods

### Study cohort and participant characteristics

A total of 60 pregnant and 20 non-pregnant women were recruited between 2020 and 2026 via the Mercy Hospital for Women (ethics managed by St Vincents Hospital Human Research Ethics Committee [HREC]), Northern Health (ethics managed by Austin Health HREC), Royal Melbourne Hospital (Melbourne Health HREC) or University of Melbourne, from which 54 and 27 blood samples, respectively, were collected. From pregnant women, 24 decidua basalis, 16 decidua parietalis, and 2 cord blood samples were collected from third-trimester term pregnancies, and 7 decidua samples were collected from first-trimester pregnancies (Supplementary Table S1). Non-pregnant participants were eligible for recruitment if they self-reported as healthy and elected to participate as a non-pregnant individual. Pregnant individuals in the first or third trimesters were eligible for recruitment if they were considered healthy and had a non-complicated pregnancy by the research midwife at the time of recruitment. The median age of pregnant and non-pregnant participants was 35 and 31 years, respectively, and was not significantly different (Supplementary Table S2). Pregnant blood samples were from first-trimester (6–12 weeks) or third-trimester (36–41 weeks) pregnancies (Supplementary Table S2). In our cohort, we did not select on any demographic variable, and only received samples from healthy pregnant and non-pregnant women. Therefore, the representativeness of our cohort might be biased towards those willing to participate in a research study or who are accessing the recruitment hospital. Robust stratification across demographics could not be performed in our manuscript as large sample numbers are needed for solid conclusions.

### Ethics

Experiments conformed to the Declaration of Helsinki Principles and to the Australian National Health and Medical Research Council Code of Practice. Written informed consent was obtained from all donors prior to the study. The studies were approved by the HRECs from Mercy Health (R14/25, R04/29 and R11/34; managed by St Vincents Hospital HREC; registered at the University of Melbourne: 1443540, 2022-23852-25877-1, 2026-31236-83504-13; third-trimester pregnancies), Austin Health (HREC/18/Austin/44; first-trimester pregnancies recruited via Northern Hospital), Melbourne Health (HREC/66341/MH-2020; registered at the University of Melbourne: 2020-20782-12450-1), and the University of Melbourne (1443389, 1955465, 2024-13344-58055-11, 2026-31236-83504-13, 2056761, 2021-14013-17863-2; non-pregnant individuals).

### Human blood processing

Whole blood was collected in heparinised blood tubes. Plasma was collected from whole blood in heparinised tubes by collecting supernatant after centrifugation (300 *g,* 10 min, 23 °C). PBMCs were isolated from heparinised whole blood through density-gradient centrifugation (Ficoll–Paque, GE Healthcare, Uppsala, Sweden). Isolated PBMCs were cryopreserved in foetal calf serum (Gibco, Sigma–Aldrich or Bovogen) with 10% DMSO (Sigma Aldrich).

### Human decidua tissue processing

Placenta processing to obtain decidua tissue was performed as described previously,^29^ with modifications for obtaining decidua parietalis and decidua from first-trimester placentae. Placentae from third-trimester pregnancies were obtained within 15 min of birth and processed within 24 h of collection. Placental lobules (cotyledons) were obtained from multiple locations on the maternal surface with basal plate and chorionic surface removed to obtain decidua basalis tissue. Meanwhile, decidua parietalis tissue was obtained by using a cell scraper on the maternal side of the chorionic membrane. Decidual tissue from first-trimester pregnancies were obtained from conceptus material from surgical terminations of pregnancies (6–12 weeks gestation) under general anaesthesia via curettage, or a combination of aspiration and curettage. Once tissues were collected, they were submerged and washed in PBS before mechanical dissociation and enzymatic digestion in RPMI 1640 medium supplemented with 2 mg/mL Collagenase D (Roche, Basel, Switzerland), 0.2 mg/mL DNAase I (Roche), 100 U/mL penicillin, 100 μg/mL streptomycin and 10 mM HEPES. Digested tissue was filtered through a 70 μm sieve and washed in RPMI 1640 containing 10% FCS. After washing, first-trimester decidua was immediately cryopreserved in FCS containing 10% DMSO, while decidual basalis tissue underwent red blood cell lysis using 0.168 M NH_4_Cl, 0.01 mM EDTA, and 12 mM NaHCO_3_ in ddH_2_O, and decidua parietalis was subjected to a density-gradient centrifugation (Ficoll–Paque, GE Healthcare) before cryopreservation in FCS with 10% DMSO.

### CMV serology

Serology was performed to determine the CMV infection status of participants. Blood plasma was subjected to a LIAISON® CMV IgG II assay (DiaSorin, Italy) to detect CMV-IgG using a chemiluminescent immunoassay according to the manufacturer’s instructions.

### Flow cytometry on fresh whole blood

All antibodies were validated for use on human cells by the manufacturers and were tested and titrated on the applicable sample type in our laboratory before use. The applicable antibody mix (Supplementary Tables S3 and S4) was added directly to 200 μL of whole blood and incubated for 30 min at ambient temperature. Fluorescently labelled samples were kept in the dark throughout the staining process. Red blood cells were lysed with 1X BD FACS™ Lysing solution (cat. no. 349202; diluted 1:10 with sterile H_2_O (Baxter, NSW, Australia)) for 10 min at ambient temperature. Samples were centrifuged (500 *g*, 5 min) and supernatant aspirated. Samples without required intracellular staining were resuspended in 1% paraformaldehyde (PFA) for 20 min at 4 °C, before washing and resuspending in MACS buffer for acquisition on a BD LSRFortessa II. Samples requiring intracellular staining were permeabilized with the eBioscience™ Foxp3/Transcription Factor Staining Buffer Set (Thermo Fisher Scientific; cat. no. 00-5523-00) for 20 min at 4 °C before washing and resuspended with intracellular antibody mix made with the Permeabilization Buffer for a further 30 min at 4 °C. The intracellular stain was washed with Permeabilization Buffer and samples were resuspended in MACS buffer for acquisition on a BD LSRFortessa II.

### Flow cytometry of decidual NK cell subsets

Cryopreserved decidua samples were thawed in pre-warmed 37 °C cRPMI containing DNase I (Roche, cat. no 4536282001) and washed in MACS buffer before resuspending in dNK subset panel surface stain mix (Supplementary Table S5). Samples were incubated for 30 min on ice, before washing twice in MACS buffer and resuspending in 1% PFA for 20 min on ice. Samples were washed, resuspended in MACS buffer and filtered through a 70 μM filter before acquiring on a BD LSRFortessa II.

### Surface proteome screening with LEGENDScreen

Human LEGENDScreen PE kit (Biolegend, cat. no. 700011; RRID: AB_3097146) antibodies (Supplementary Table S6) were reconstituted according to the manufacturer’s protocol. All antibody incubations were performed for 30 min, on ice and in the dark unless otherwise stated. PBMCs and placenta single-cell suspension samples were thawed with Benzonase nuclease (Merck Life Science Pty Ltd cat. no. E8263-25KU) before staining in 6 × 10^6^ cell aliquots for optimal labelling. Individual samples were barcoded with combinations of Cell Trace Violet (CTV) and/or anti-CD45-biotin plus secondary labelling with fluorochrome-conjugated streptavidin to allow pooling of donors for immune analysis, adapted from.^30^ Samples requiring CTV labelling were resuspended in 1 μM CTV (ThermoFisher Scientific, VIC, Australia) for 13 min at 37 °C (gentle mix every 3–4 min). CTV-labelled samples were washed, and all samples stained with 1:100 anti-CD45-biotin (Biolegend). Samples were washed twice in MACS buffer (0.5% BSA, 2 mM EDTA in PBS) and resuspended in donor-specific barcode mixes containing streptavidin-BUV395, -BUV496, and/or -BUV805 (Supplementary Table S7). Following washing (x2), samples were stained with MR1-5-OP-RU-BV421 tetramer (1:100) for 30 min at room temperature. Samples were washed twice and resuspended in the backbone panel to determine immune cell populations (Supplementary Table S8). Samples were washed, pooled and evenly distributed across the Human LEGENDScreen PE kit, where cells were stained per the manufacturer’s protocol. Samples were resuspended in MACS buffer and acquired on the Cytek Aurora 5-laser flow cytometer.

### Surface proteome data analysis

LEGENDScreen flow cytometry data were analysed by first deconvoluting individual samples with donor barcode fluorophores. Individual sample gates were exported as new.fcs files. Manual gating was performed on individual marker-PE wells to determine frequencies of marker-PE^+^ cells for each immune cell subset (Supplementary Fig. S1A). LEGENDScreen markers overlapping with the backbone panel were not included in differential expression analysis due to potential competitive binding, and only markers with a frequency of ≥10% in at least one sample were analysed for frequencies. Uncorrected Mann–Whitney U tests were used to compare non-pregnant and pregnant blood, and an uncorrected Wilcoxon test was used to compare paired pregnant blood and decidua. To provide further insight on markers to validate in NK cells, we also performed a moderated t-test using limma^31^ on log transformed marker-PE^+^ and correlated the moderated t-statistic with our uncorrected p-value (Supplementary Fig. S1B and C). To reduce the number of false-positive hits, we utilised a combination of statistical significance, a log_2_ fold-change (log_2_FC) > 0.5 and a requirement of 20% of cells expressing in at least one group per comparison.

### scRNAseq of blood and decidua basalis NK cells

PBMCs and decidua basalis single-cell suspension samples were thawed, washed and resuspended in 50 μL of MACS buffer. Samples were sequential stained at 4 °C and in the dark with 5 μL of TruStain FcX (Biolegend, cat. no. 422301) incubated for 10 min; fluorescent monoclonal antibody mix (Supplementary Table S9) for 30 min; 20 μL of TotalSeq™-C Human Universal Cocktail (Biolegend, cat. no. 399905; RRID: AB_2876728) and 2 μL of one of the TotalSeq-C anti-human Hashtag antibodies 1–6 (cat. nos. 394661 (RRID: AB_2801031), 394663 (RRID: AB_2801032), 394665 (RRID: AB_2801033), 394667 (RRID: AB_2801034), 394669 (RRID: AB_2801035), 394671 (RRID: AB_2820042)) for 30 min. Samples were washed, resuspended with MACS buffer before NK cells were sorted into RPMI with 10% foetal calf serum on a Sony SY3200 cell sorter (Sony Biotechnology, Illinois, USA). Sorted cells were counted and mixed into 10× Chromium reactions before being loaded into Chromium Next GEM Chip K (PN 2000182, CA, USA) and run on the Chromium Controller iX according to manufacturer’s instructions (Chromium Next GEM Single Cell 5’ v2 (Dual Index) with Feature Barcoding technology; Revision D). Gene expression (GEX) and feature barcode (CITE-seq) libraries were generated with Chromium Next GEM Single-Cell 5′ v2 kits according to manufacturer’s instructions. Sequencing was performed using an Illumina NovaSeq at a read length of 26 × 90 bp.

### Single-cell RNA sequencing data analysis

Raw sequencing data were processed with Cell Ranger v7.1.0 (10× Genomics). Donors were demultiplexed based on differences in single nucleotide polymorphisms using cellsnp-lite v1.2.2 and vireo v0.2.3.^32^^,^^33^ Gene expression (GEX) and Feature Barcode (CITE-seq) matrices were analysed with the Seurat package v4.3.0.1.^34^ Gene expression data were filtered for high-quality cells by removing cells with more than 10% mitochondrial mRNA, and cells with <500 or >5000 genes. CITE-seq data were filtered by removing cells with total reads outside 3 × the median absolute deviation from the median library size. Sample demultiplexing was performed using the hashedDrops function from DropletUtils v1.28.1.^35^ CITE-seq was normalised against isotypes using the dsb package v1.0.3.^36^ The RunHarmony function from the Harmony R package v0.1.1^37^ was applied to RNA and CITE-seq level data for donor/batch integration before running a WNN-UMAP. To integrate RNA and protein level data, weighted-nearest-neighbour (WNN) Uniform Manifold Approximation Projection (UMAP) was performed using FindMultiModalNeighbors^34^ using the first 25 and 18 principal components for RNA and protein, respectively. Clusters were identified using the FindNeighbors and FindClusters using a resolution of 0.4 and the Smart Local Moving algorithm for clustering. The FindMarkers function from Seurat was used to identify differentially expressed genes (DEGs) and proteins (DEPs) between clusters to help in assigning them to a relevant NK cell subset. Group analysis between pregnant and non-pregnant, or pregnant peripheral and decidual NK cells was performed using a pseudo-bulk approach after removing unwanted variance (RUV). Unwanted variation was estimated with RUV-4 algorithm implemented in the ruv R package v0.9.7^38^ with k = 4. Only cells from clusters 2, 3 and 8 were considered true decidual NK cells for group analyses. Pseudo bulking was performed by aggregating counts across groups, donors, and Harmony clusters. Differential expression (DE) analysis between groups was performed using the edgeR package v 3.42.4.^39^ Data were fitted with a negative binomial generalised log-linear model implemented in glmFit function.^40^ Genes were considered differentially expressed between groups if they achieved an FDR <5% after likelihood ratio tests. Differential expression (DE) analysis of adaptive-like NK subsets, and *SOCS1* subsets was performed using the Seurat FindMarkers function. Gene set enrichment analysis was performed with clusterProfiler v4.13.4^41^ and enrichplot v1.25.6^42^ packages.

### Integration of public scRNAseq datasets

First-trimester dataset was downloaded from ArrayExpress (E-MTAB-6701 and E-MTAB-6678). Third trimester dataset was obtained via the National Institutes of Health dbGaP repository (phs001886.v5.p1).

The first-trimester dataset was loaded as a Seurat object from the published 10× raw count matrix and its corresponding metadata. Counts were log-normalised and variable features were identified; T-cell and B-cell receptor variable/joining segment genes (matching TRA/TRB and IGK/IGL/IGH V and J genes) were removed from the variable-feature set. The data was scaled, principal component analysis (PCA)computed, and cells were embedded by UMAP and clustered (shared-nearest-neighbour graph followed by Louvain clustering at resolution 0.2) using the first 20 principal components.

For third trimester dataset, raw sequencing reads were downloaded and aligned to the GRCh38 (hg38) human reference using CellRanger (v9.0.0) count to generate per-sample filtered feature–barcode matrices. Quality-control thresholds were set for each sample based on median plus three median absolute deviation. Cells were retained that expressed between 200 and (median + 3 × MAD) detected genes, had between 500 and (median + 3 × MAD) total UMI counts, and a mitochondrial content (percentage of reads mapping to MT-genes) below (median + 3 × MAD). Filtered counts were log-normalised and the 2000 most variable genes were identified. Given the size of the dataset, dimensionality reduction and clustering were performed using the Seurat sketch-based workflow. A representative subsample of 7000 cells was selected by leverage-score. Within this sketch, variable features were re-selected, the data were scaled, and PCs computed. Cells were clustered on the first 20 PCs (shared-nearest-neighbour graph followed by Louvain clustering at resolution 0.5) and embedded by UMAP. The sketch-derived PCA and cluster assignments were then projected onto the full dataset, and a UMAP of all cells was computed from the projected principal components. Cells were annotated with CellTypist (v1.6.3)^43^^,^^44^ using the Human Placenta Decidua reference model. Per-cell predictions were imported and a single consensus label was assigned to each projected cluster by majority voting.

To analyse expression of *SOCS1* and *AREG* across datasets, a pseudobulk differential-expression analysis was performed. The two datasets were merged and labelled by timepoint and donor. For each broad cell type present in both datasets, raw counts were summed across cells to yield one pseudobulk profile per donor × timepoint. Pseudobulk samples derived from fewer than 20 cells were discarded, and cell types with fewer than two donors per timepoint were excluded. Pseudobulk matrices were analysed with edgeR (v.4.4.2) and limma (v.3.62.2): libraries were TMM-normalised, lowly expressed genes were removed and cell types retaining fewer than 100 genes were skipped.

### Receptor-ligand interaction analysis

To enable cross-dataset ligand-receptor comparison, the fine-grained cell-type labels of both interface datasets were collapsed into a shared set of broad lineages. The in-house NK dataset was then merged into each interface dataset and the combined objects were log-normalised, providing a shared, high-resolution NK annotation across both timepoints.

Ligand–receptor interactions were inferred separately for each timepoint using LIANA (v.1.14),^45^ with the harmonised broad-lineage/NK-subtype labels as the grouping variable. LIANA was run across its panel of scoring methods and a consensus interaction ranking (aggregate rank) was computed by robust rank aggregation; this aggregate rank was used as the interaction-strength metric. Analysis focused on interactions in which an NK subtype (dNK2, dNK3 or dNK4) acted as the ligand-expressing (sending) or receptor-expressing (receiving) partner.

### Protein-protein interaction network

Protein-protein interaction analysis and network was generated in Cytoscape v3.10.0^46^ using the STRING *Homo sapiens* database. The genes of interest (*AREG* and *SOCS1*) were queried with a maximum additional interactor of 30, and a confidence score cutoff of 0.7.

### PBMC cytokine stimulation

PBMCs were stimulated with IL-12 (10 ng/mL) and IL-18 (100 ng/mL), or IFN-γ (100 ng/mL) for 6, 24, or 48 h. When intracellular cytokine staining was performed, brefeldin A (GolgiPlug, BD Biosciences cat. no. 555029) and monensin (GolgiStop, BD Biosciences cat. no. 554724) were added. At the endpoint, supernatants were collected from separate wells that did not receive brefeldin A or monensin, for use in the LEGENDplex assay (described below).

### Cytokine measurements with LEGENDplex

Levels of IL-1β, IFN-α2, IFN-γ, TNF, MCP-1, IL-6, IL-8, IL-10, IL-12p70, IL-17A, IL-18, IL-23, and IL-33 in stimulated PBMC culture supernatants were measured using the LEGENDplex Human Inflammation Panel-1 kit (BioLegend, cat. No. 740809). The assay was performed according to the manufacturer’s instructions. Samples were acquired on a BD LSRFortessa II and analysed with QOGNIT LEGENDplex software.

### Immunoblotting for SOCS1

PBMCs at baseline or after stimulation with IL-12/IL-18 or IFN-γ (described above) were harvested and pellets snap frozen before lysing with sample buffer. Equivalent number of cells based on post-harvest cell counts were loaded onto a 20-well NuPAGE 4–12% Bis-Tris Midi Gel (Invitrogen, cat. no. WG1402). Samples were transferred to nitrocellulose membrane at 100 V for ∼100 min at 4 °C. Membranes were blocked before probing for SOCS1 (Cell Signalling Technologies, cat. no CST#68631) or β-actin-HRP (Santa Cruz, cat. no. 47778; RRID: AB_626632) for 1 h at ambient temperature. Anti-rabbit-HRP (SouthernBiotech, cat. no. 4030-05; RRID: AB_2687483) was then applied to SOCS1 blots for an additional hour. Samples were developed with 2-component enhanced chemiluminescence (Millipore, cat. no. WBULS0100).

### Statistics

All datapoints shown are from distinct samples. Sample sizes were not pre-determined using statistical methods but instead were determined by availability of samples from pregnant and non-pregnant women of reproductive age in our cohorts. Experiments were not blinded and datasets were assumed to be non-normal and non-parametric tests were performed. Statistical significance for comparisons between more than two unpaired groups was determined with a Kruskal–Wallis ANOVA with Dunn’s multiple comparisons. Statistical significance for comparisons between more than two paired groups was determined with a Friedman test with Dunn’s multiple comparisons. Statistical significance of univariate comparisons between two unpaired groups was determined with Mann Whitney *U* tests, while a Wilcoxon test was used for univariate comparisons between two paired groups. When comparing different tissues (e.g., decidua basalis, decidua parietalis, blood) from the same individuals, a paired non-parametric test was used.

Multiple comparisons have been corrected for where appropriate throughout the manuscript. One exception is the LegendScreen dataset as the sample size was limited, and p-value correction resulted in increased type II error (false-negatives). To mitigate potential false-positive hits from LegendScreen, we performed follow-up experiments in increased sample sizes as validation.

### Role of funders

Funders were not involved in the study design, data collection, data analyses, interpretation or writing of the report.

## Results

### Pregnancy alters surface proteome of lymphoid and myeloid immune subsets

To define the surface proteome of lymphoid and myeloid immune subsets during pregnancy in-depth, we screened >350 cell-surface proteins using a ‘Massively Parallel Cytometric’ approach (Fig. 1A). We labelled pregnancy-matched maternal peripheral blood mononuclear cells (PBMCs), cells recovered from decidua basalis and umbilical cord blood, and PBMCs from non-pregnant women, with unique fluorescent barcodes and a backbone panel of lineage and activation markers (Supplementary Tables S7 and S8). Samples were multiplexed and labelled with PE-conjugated mAbs for parallel screening. Individual samples were deconvoluted based on fluorescent barcodes and manually gated to identify key immune populations: NK cells, CD8^+^ T cells, CD4^+^ T cells, γδ T cells, B cells, and monocytes (Fig. 1B, Supplementary Fig. S1A). Across the six immune cell subsets, 110 unique proteins were differentially expressed between pregnant and non-pregnant groups (Fig. 1C; Supplementary Fig. S2).

**Fig. 1:**
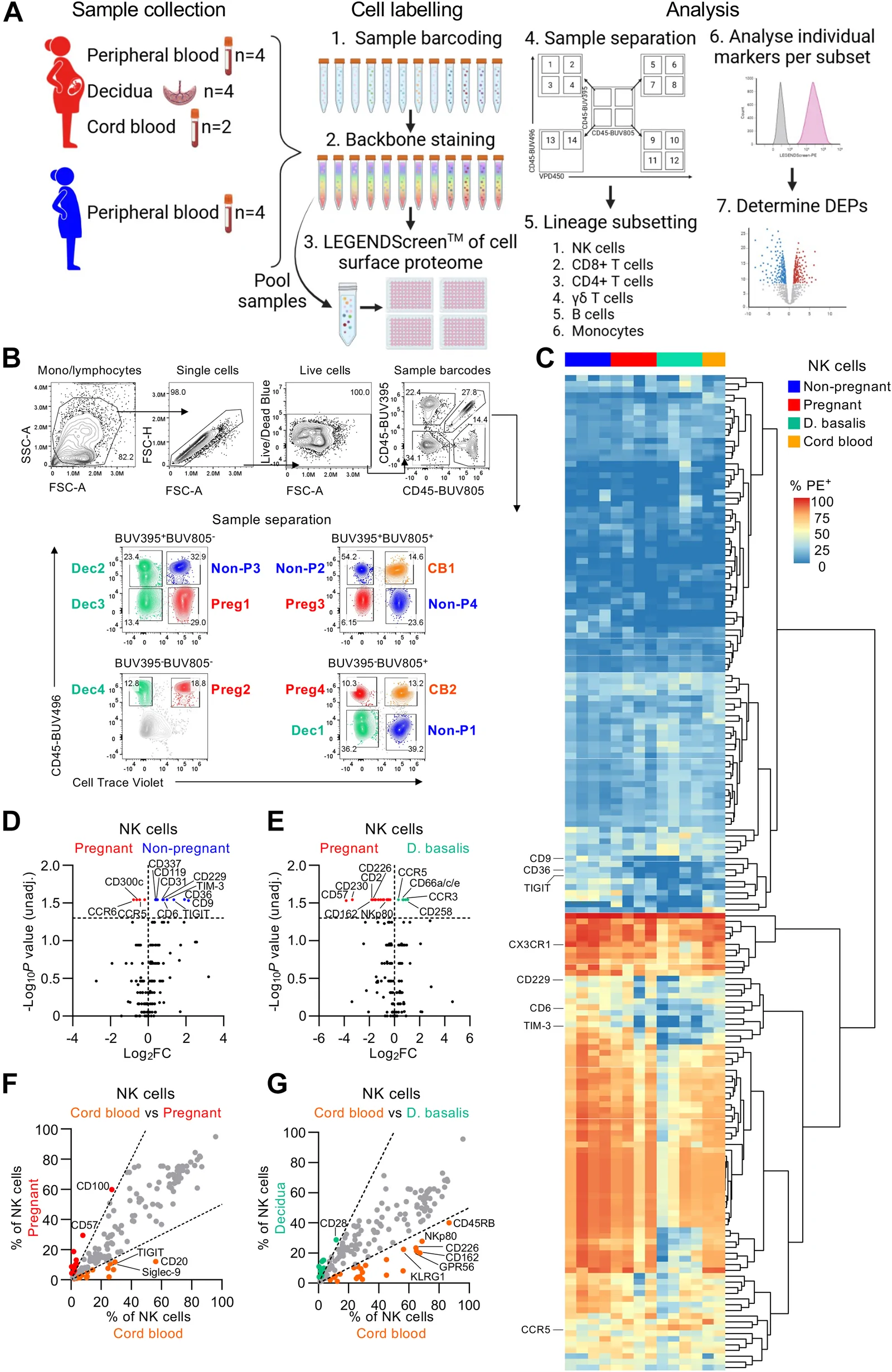
**Surface proteome profiling of immune cells from pregnant and non-pregnant women peripheral blood, cord blood and decidua.** Each panel contains data from pregnant (n = 4) or non-pregnant (n = 4) peripheral blood, decidua (n = 4), and cord blood (n = 2) samples. (A) Schematic representation of the massively parallel cytometry experiment workflow. (B) Representative FACS plots of the gating strategy to deconvolute individual samples based on unique fluorescent barcoding. (C) Heatmap with hierarchical clustering of columns showing frequencies of marker-PE expression on NK cells from pregnant and non-pregnant peripheral blood, decidua, and cord blood. Markers expressed on >10% of cells in at least one sample were included for analysis (174 markers). (D and E) Volcano plots depicting differential protein expression in non-pregnant vs. pregnant blood NK cells (D) and decidual vs. pregnant blood NK cells (E). Volcano plot horizontal dashed line indicates log_10_ p-value cutoff of 1.3 (equivalent to p < 0.05 from a Mann-Whitney U test or Wilcoxon signed-rank test). (F and G) Scatter plot of average percent of cells expressing for cord blood vs. pregnant peripheral (F) and cord blood (n = 2) vs. decidual (n = 4) (G) NK cells. Scatter plot dashed lines indicate a log_2_FC cutoff of 1.

Given the important roles NK cells play in pregnancy, we began by focusing our analyses on the differential protein expression on NK cells between pregnant and non-pregnant blood. We identified increased expression of migration markers (CCR5, CCR6) and the activating receptor CD300c, but reduced frequencies of NK cells expressing markers of exhaustion/activation (TIGIT, TIM-3, CD119/IFNGR1, CD337/NKp30, CD229/SLAMF3), scavenger receptors (CD6, CD36), tetraspanin CD9, and the adhesion molecule CD31 in pregnant blood compared to non-pregnant blood (Fig. 1D). Of these, CD9, CD36, TIGIT, and TIM-3 frequencies were reduced by more than 2-fold (log_2_ fold-change (log_2_FC) > 1) (Fig. 1D; Supplementary Table S10). CD9 was consistently decreased across CD8^+^, CD4^+^, and γδ T cells, while CD119/IFNGR1 was decreased on γδ T cells and B cells from the pregnant blood (Supplementary Fig. S3A and Table S10). Of the markers increased on NK cells from pregnant individuals, CD195/CCR5 was also increased on CD8^+^ T cells (Supplementary Fig. S3A). Within the T cell compartment, there were 17, 3, and 13 differentially expressed proteins across CD8^+^, CD4^+^, and γδ T cells, respectively. Notably, migration/adhesion markers CD195/CCR5, CX3CR1, and CD11c/ITGAX were increased on CD8^+^ T cells in pregnancy, while co-stimulatory molecules CD26 and CD28 were decreased (Supplementary Fig. S3A). In contrast to NK cells and CD8^+^ T cells, there was lower CD195/CCR5 expression on γδ T cells, with decreases in CD218a/IL18Ra, CD278/ICOS, CD94, CD226/DNAM-1, and CD183/CXCR3 expression also observed in pregnancy (Supplementary Fig. S3A; Supplementary Table S10).

We next investigated differences between pregnant blood and decidua basalis from pregnancy-matched samples. Twenty-three differentially expressed proteins were observed on NK cells between pregnant blood and decidua, with adhesion molecules (CD99, CD230/prion), activation markers (NKp80, CD226/DNAM-1, CD2), scavenger receptor CD6, anti-migration/inhibitory marker GPR56, migration marker CD162/P-selectin glycoprotein ligand-1 (PSGL-1), and the adaptive-like NK cell marker CD57 being higher in the blood, while only CD258/LIGHT was more than 2-fold higher (log_2_FC > 1) in decidual NK (dNK) cells (Fig. 1E, Supplementary Table S11). Frequencies of NK cells expressing chemokine receptors CD193/CCR3 and CD195/CCR5 were also higher on dNK but with a lower fold-change (Supplementary Table S11). All three proteins (CD193/CCR3, CD195/CCR5, and CD258/LIGHT) were also increased on decidual CD8^+^ and CD4^+^ T cells, while CD195/CCR5 was shared with B cells. CD162/PSGL-1, CD99, and CD230/prion were higher across all lymphocyte subsets from the peripheral blood during pregnancy, indicating their expression is of importance for circulating lymphocytes, but not monocytes (Supplementary Fig. S3B; Supplementary Table S11). Furthermore, like dNK cells, there was less expression of CD57 on peripheral γδ T cells, suggesting there may be less adaptive-like or terminally-differentiated NK and γδ T cells within the tissue (Supplementary Fig. S3B; Supplementary Table S11). Markers shared between CD4^+^ and CD8^+^ T cells in the decidua included migration/adhesion markers CD193/CCR3, CD195/CCR5, CD11c, and CXCR7, and Fc-γ receptors (FcRs) CD32 and CD64 (Supplementary Fig. S3B; Supplementary Table S11).

We also analysed differentially expressed markers on NK cells in the relatively immunologically-naïve cord blood. In comparison to pregnant peripheral blood, NK cells in the cord blood had increased CD9 and inhibitory/exhaustion markers Siglec-9 and TIGIT (Fig. 1F; Supplementary Table S12). CD9 and Siglec-9 were also increased on cord blood monocytes, and CD9 on cord blood B cells (Supplementary Fig. S3C; Supplementary Table S12). Lower CD57 expression was observed on cord blood NK cells and γδ T cells compared to pregnant blood, an indication of a lack of adaptive-like or terminally-differentiated cells in the immunologically naïve neonate (Supplementary Fig. S3C; Supplementary Table S12). When compared to dNK cells, 21 markers were increased on cord blood NK cells, including CD360/IL-21R, CD24, CD230, and CD162/PSGL-1, which were also increased on cord blood CD8^+^, CD4^+^ and γδ T cells (Fig. 1G; Supplementary Fig. S3D; Supplementary Table S13). Furthermore, inhibitory molecule CD200 was increased in all cord blood T cell subsets when compared to pregnant blood or decidual T cells, and adhesion molecule CD49a was increased on cord blood CD4^+^ and CD8^+^ T cells as compared to pregnant blood (Supplementary Fig. S3C and D; Supplementary Tables S12 and S13). Markers increased on cord blood B cells compared to both pregnant blood and decidua included differentiation marker CD10, chemokine receptors CD192/CCR2 and CD193/CCR3, B-1 cell associated markers CD5 and CD148 (Supplementary Fig. S3C and D; Supplementary Tables S5 and S6). There were 66 proteins increased on cord blood monocytes compared to pregnant blood and 34 when compared to the decidua. The top 5 differentially expressed markers increased on cord blood monocytes were CD2, CD15, CD41, CD200R, and CD354 in comparison to pregnant blood, of which CD2, CD15, and CD41 were also within the top 5 when compared to decidual monocytes (Supplementary Fig. S3C and D; Supplementary Tables S12 and S13). Taken together, surface proteome analysis with LEGENDScreen revealed numerous differentially expressed proteins in lymphocyte and myeloid subsets during pregnancy. To gain further insight on the impact of pregnancy has on immunity, we focused on NK cells to link differentially expressed proteins and transcriptomic profiles using single-cell RNA sequencing.

### NK cell heterogeneity in peripheral blood and decidua

NK cell features in healthy non-pregnant peripheral blood and across tissues are well-defined. From a pregnancy perspective, multiple studies have investigated decidual or uterine NK cells, but there remain limited data exploring peripheral blood NK cells in pregnancy, as compared to non-pregnant individuals, at the single-cell resolution. Therefore, we used scRNAseq with cellular indexing of transcriptomes and epitopes (CITE-seq) to assess RNA expression as well as 130 surface proteins via antibody-derived tags (ADTs) to analyse CD56^+^CD3^−^ NK cells from the blood of pregnant and non-pregnant individuals, and pregnancy-matched decidua basalis tissue (Fig. 2A; Supplementary Figs. S4 and S5). RNA and protein expression data were used to generate a weighted nearest neighbour uniform manifold approximation projection (WNN-UMAP) of NK cells (Fig. 2B and C). Following quality control, six unsupervised NK cell clusters were generated from peripheral blood (clusters 0, 4, 6, 7, 9, 12) and seven clusters from decidua basalis cells (clusters 1, 2, 3, 5, 8, 10, 11) (Fig. 2C and D).

**Fig. 2:**
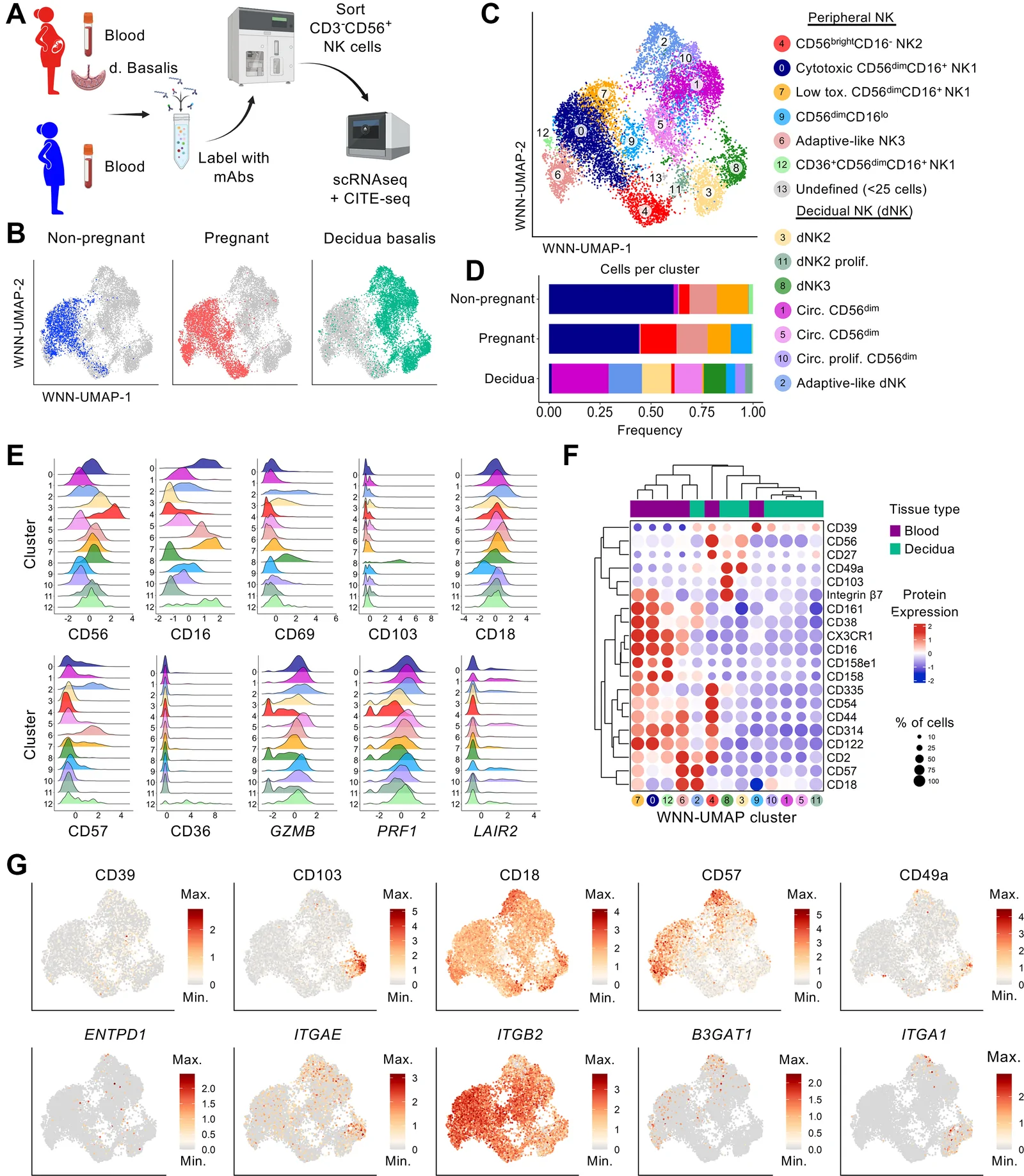
**Single cell resolution of NK cell transcriptomes and proteomes in pregnancy.** Each panel contains data from pregnant (n = 3) or non-pregnant (n = 2) peripheral blood or decidua (n = 3) samples. (A) Schematic overview of the experimental approach for scRNAseq on NK cells from pregnant and non-pregnant peripheral blood, and the decidua. (B) WNN-UMAP with NK cells coloured by pregnant, non-pregnant or decidua groups. (C) WNN-UMAP of NK cells based on protein and gene expression. (D) The proportion of cells in each WNN-UMAP cluster per group. (E) Histograms depicting the expression levels of selected proteins and genes used to define the WNN-UMAP clusters. (F) Hierarchically clustered dot plot of 20 selected proteins contributing to distinct NK cell phenotypes observed in each WNN-UMAP cluster. (G) WNN-UMAPs of dNK markers CD39/*ENTPD1*, CD103/*ITGAE*, CD18/*ITGB2*, CD57/*B3GAT1*, and CD49a/*ITGA1* at the protein (top row) and RNA (bottom row) level.

Clusters were manually annotated using protein expression of canonical NK cell markers CD56 and CD16 to define CD56^bright^CD16^−^ (clusters 3, 4, 8, 11) and CD56^dim^CD16^+^ (clusters 0, 1, 2, 5, 6, 7, 9, 10, 12), in addition to tissue-residency/dNK markers (CD69, CD103, and CD18), senescence marker CD57, and CD36, *GZMB*, *PRF1*, and *LAIR2* (Fig. 2E). Furthermore, 20 surface proteins and 75 genes previously associated with peripheral blood or dNK subsets^25^^,^^47^^,^^48^ were used to further define and validate the NK cell subset associated with each cluster (Fig. 2F; Supplementary Fig. S4; Supplementary Table S14). Based on this, circulating NK cells from the blood were cytotoxic CD56^dim^/NK1 (clusters 0, 7, 12), CD56^bright^/NK2 (cluster 4), or adaptive-like NK3 (cluster 6). Notably, cluster 9 did not fall into the peripheral NK1-3 subsets, was CD56^dim^CD16^lo^, and only contained NK cells from one pregnant participant’s blood and decidua (Fig. 2B and C; Supplementary Fig. S4C).

Decidual NK cell clusters were assigned using markers described by Vento-Tormo et al. where possible^25^ (Fig. 2C–F; Supplementary Table S14). dNK clusters 2, 3, 8, and 11 expressed tissue-residency marker CD49a and were selected for downstream analysis, while clusters 1, 5, and 10 were likely contaminating cells from the circulation due to lack of CD49a expression (Fig. 2F and G). The dNK1-3 subsets were originally described in first-trimester decidua, however our dataset of third-trimester decidua basalis was lacking dNK1 cells, defined by CD39/*ENTPD1* expression (Fig. 2F and G). Meanwhile, dNK2 (CD18^+^CD39^−^) and dNK3 (CD103^+^) subsets were detectable in clusters 3, 8, and 11 (Fig. 2F and G). Interestingly, the dNK cells within cluster 2 uniquely expressed CD57, a marker of an adaptive-like subset which has not been previously described in dNK cells (Fig. 2E–G).

### A unique adaptive-like dNK population is enriched in cytomegalovirus seropositive pregnancies

Both peripheral blood NK and dNK cells formed clusters of adaptive-like NK cells (clusters 6 and 2, respectively; Fig. 2). Adaptive-like NK cells in the blood have been described as expressing of *KLRC2*, *B3GAT1*/CD57, and *LAG3*/CD223 (LAG-3),^47^^,^^48^ and notably expansion of this subset is associated with a history of cytomegalovirus (CMV) exposure.^47^ While adaptive-like NK cells are well-described in the blood, the existing decidual dNK1-3 subsets are not associated with features of an adaptive-like subset. Therefore, to validate the presence of a fourth adaptive-like “dNK4” subset in the decidua, we used flow cytometry for key dNK subset markers CD39 and CD103, with the addition of adaptive-like marker CD57, and determined whether it was associated with pregnancy trimester or the decidua basalis or decidua parietalis compartments (Supplementary Fig. S6A). After gating on CD49a^+^ tissue-resident dNK cells, CD39 and CD103 were used to distinguish the dNK1 and dNK3 subsets, respectively, then CD39^−^CD103^−^ cells (dNK2) were bifurcated by CD57 expression, supporting the presence of an additional tissue-resident adaptive-like CD57^+^ dNK4 subset (Fig. 3A). CD57^+^ dNK4 cells were detected in the first-trimester decidua and third-trimester decidua basalis and parietalis compartments (Fig. 3A). CD57 expression was unique to dNK4, with minimal expression in dNK1 and dNK3 subsets (mean 2.9% and 3.4%, respectively) and only in few samples (Fig. 3B).

**Fig. 3:**
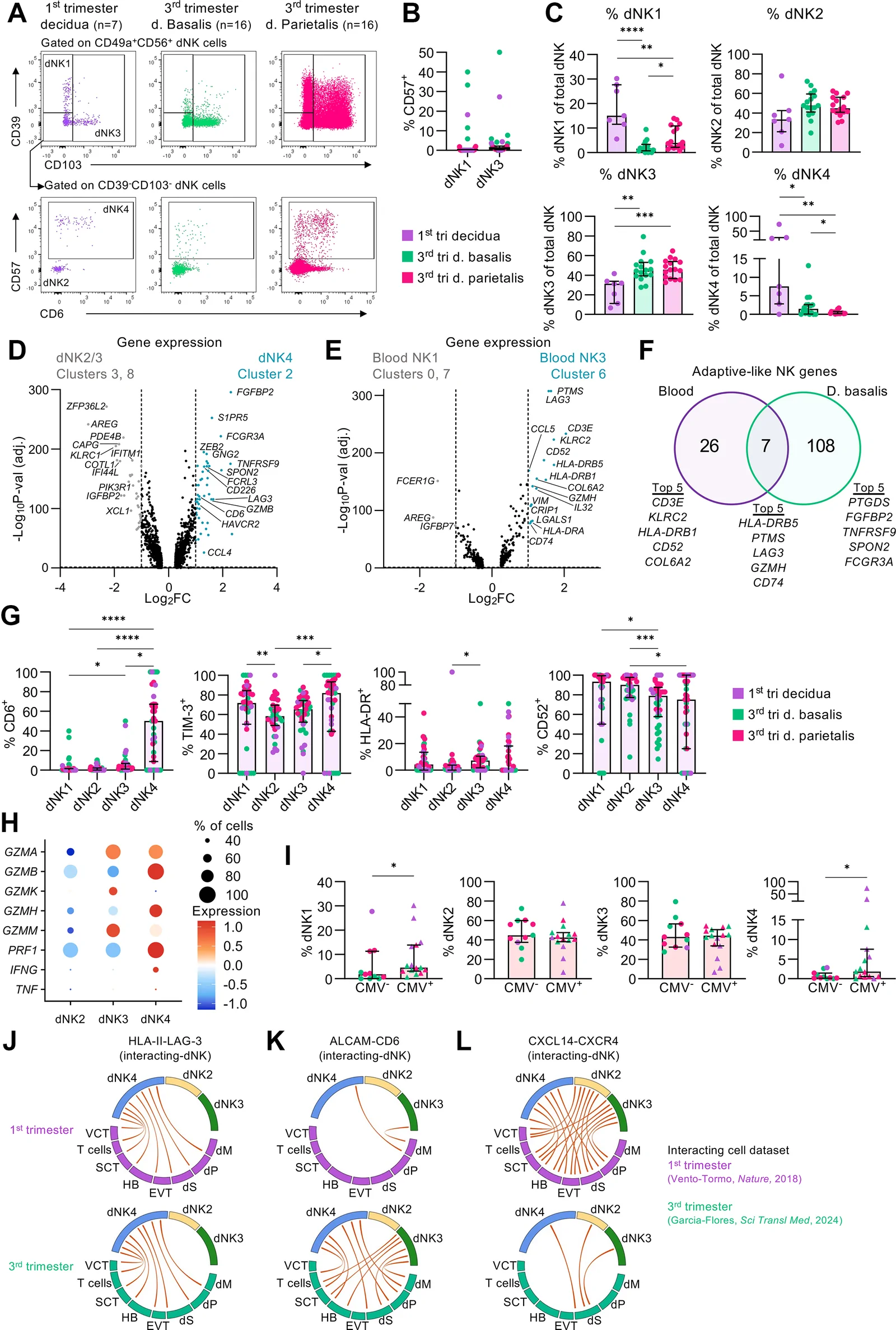
**Dynamics of dNK subsets in early and late pregnancy.** (A) Overlayed FACS plots from first-trimester decidua (n = 7) and third-trimester decidua basalis (n = 16) and parietalis (n = 16) depicting CD39 and CD103 (top row) and CD57 vs. CD6 expression (bottom row) on CD49a^+^CD56^+^CD3^−^ dNK cells to identify dNK1-4 subsets. (B) Frequencies of CD57^+^ dNK1 and dNK3 cells within first-trimester decidua (n = 7) and third-trimester decidua basalis and parietalis (n = 16). (C) Frequencies of dNK1-4 subsets within first-trimester decidua (n = 7) and third-trimester decidua basalis and parietalis (n = 16). A Mann–Whitney U test was performed between first-trimester and third-trimester samples to determine significance. A Wilcoxon signed-rank test was used to determine statistical significance between decidua basalis and parietalis tissues. (D) Volcano plot of differential gene expression between dNK2 and 3 and adaptive-like dNK4 subsets. (E) Volcano plot of differential gene expression between peripheral CD56^dim^/NK1 and adaptive-like CD56^dim^/NK3 subsets. (F) Venn diagram depicting the unique and overlapping genes between blood and decidual adaptive-like NK cells, with top 5 genes based on fold-change listed. (G) Frequencies of CD6^+^, TIM-3^+^, HLA-DR^+^, and CD52^+^ cells across the dNK1-4 subsets. A Wilcoxon signed-rank test was used to determine statistical significance. (H) DotPlot depicting percent of cells expressing and scaled expression levels of *GZMA, GZMB, GZMK, GZMH, GZMM, PRF1, IFNG,* and *TNF* within dNK2, 3 and 4 subsets. (I) Frequencies of dNK1-4 subsets in decidua from sero-negative (CMV^−^; n = 11) and sero-positive (CMV^+^; n = 15) pregnancies. A Mann–Whitney U test was used to determine statistical significance. (J–L) Circos plots depicting receptor-ligand interactions between dNK2, 3 and 4 subsets and first- or third-trimester decidual cells for LAG-3-HLA-II (J), CD6-ALCAM1 (K), and CXCR4-CXCL14 (L). VCT: villous cytotrophoblast, SCT: syncytiotrophoblast, HB: Hofbauer cells, EVT: extra villous trophoblast, dS: decidual stroma, dP: perivascular cells, dM: decidual macrophages. (D and E) Horizontal dashed lines depict statistical significance threshold, vertical dashed lines indicate the log_2_FC cutoff. p-values from Mann-Whitney U test or Wilcoxon signed-rank test denoted as: ∗p < 0.05, ∗∗p < 0.01, ∗∗∗p < 0.001, ∗∗∗∗p < 0.0001.

Having established a fourth dNK subset, we subsequently sought to understand the dynamics of the dNK1-4 subsets over the course of pregnancy. Frequencies of dNK1 and dNK4 were highest in first-trimester decidua when compared to both the decidua basalis and parietalis compartments from third-trimester pregnancies (p < 0.0001 and p < 0.01, respectively; Mann–Whitney U test), providing further evidence that the dNK1 subset has declined by the third pregnancy trimester (Fig. 3C). Notably, there was a bimodal distribution of dNK4 frequencies, with three first-trimester pregnancies having frequencies >25%, and four having frequencies <7.5% (Fig. 3C). When comparing decidua basalis and parietalis compartments, we observed significantly higher frequencies of dNK1 cells in the parietalis (p < 0.05; Wilcoxon signed-rank test), while adaptive-like dNK4 were increased in the basalis (p < 0.05; Wilcoxon signed-rank test), although both of these subsets make up a relatively minor population in the third-trimester (Fig. 3C). Conversely, higher frequencies of dNK3 were observed in third-trimester decidua basalis and parietalis (p < 0.01 and p < 0.001, respectively; Mann–Whitney U test), while dNK2 frequencies were consistent across pregnancy trimesters and decidual compartments (Fig. 3C). The skewing of dNK subset frequencies over the course of pregnancy could reflect different functional requirements between early vs. late pregnancy.

To further define the unique dNK4 subset, we examined differentially expressed genes from our third-trimester scRNAseq dataset between dNK4 and dNK2/3. Key genes upregulated in dNK4 included *CD226 (DNAM-1)*, *LAG3* and *HLA-DRB5,* while interferon stimulated genes (*IFITM1, IFI44L*) and *AREG* were downregulated (Fig. 3D; Supplementary Table S15). *AREG* (amphiregulin) is known for its role in wound healing, and is associated with circulating CD56^bright^/NK2. We observed that adaptive-like circulating and dNK cells had the lowest *AREG* expression across all NK cell clusters and groups (Supplementary Fig. S6B and C). Additionally, we determined the overlap in genes upregulated in dNK4 and adaptive-like circulating NK3 subset in the blood to select shared and unique markers to measure across dNK subsets via flow cytometry (Fig. 3D–F; Supplementary Tables S15 and S16). From this, we selected to measure CD6, TIM-3, HLA-DR and CD52 expression across dNK subsets via flow cytometry. Most striking was the frequency of CD6^+^ cells within the dNK4 subset, which was ∼7-23-fold higher than the dNK1-3 subsets (Fig. 3G). Interestingly, in the context of peripheral blood NK cells, CD6 is known to be expressed on a subset of CD56^dim^ NK cells, of which direct triggering can be linked to cytokine and chemokine secretion.^49^ Meanwhile, TIM-3 expression was higher in both dNK1 (p < 0.01; Wilcoxon signed-rank test) and dNK4 (compared to dNK2: p < 0.001, compared to dNK3: p < 0.05; Wilcoxon signed-rank tests), while HLA-DR was higher in dNK3 than dNK2 (p < 0.05; Wilcoxon signed-rank test), and CD52 was lower in dNK3 (p < 0.001; Wilcoxon signed-rank test) and dNK4 (p < 0.05; Wilcoxon signed-rank test) in comparison to dNK2 (Fig. 3G). To gain additional insight on the functionality of dNK4, we assessed expression in our scRNAseq dataset of RNA encoding cytotoxic granules in dNK2, 3 and 4 subsets. dNK4 had higher expression of *GZMB, GZMH, PRF1*, while dNK2 had higher *GNLY*, and dNK3 higher *GZMM* and *GZMK* (Fig. 3H).

Furthermore, given that increased frequencies of adaptive-like NK cells in the blood are associated with CMV exposure history, and CMV reactivation contributes to the global burden of pregnancy complications, with up to 42% of pregnant women developing non-primary CMV infections,^50^ we next determined whether there was an enrichment of dNK4 in the decidua of individuals with a history of CMV based on serology. In CMV-seropositive pregnancies, there were higher frequencies of dNK1 (p < 0.05; Mann–Whitney U test) and dNK4 (p < 0.05; Mann–Whitney U test) subsets, albeit at a relatively low magnitude (Fig. 3I). While the exact role of adaptive-like dNK4 remains undefined, it may play a role in protecting against vertical transmission of CMV. However, on the contrary, an inflation of dNK4 could have a detrimental impact on the establishment of pregnancy due to increased markers of exhaustion (*HAVCR2*/TIM-3 and LAG-3) and a lack of key molecules implicated in decidualisation (e.g., *AREG*), warranting further investigation.

To gain a better understanding of the role of the dNK2, 3 and 4 subsets in the decidua, receptor-ligand interaction analysis was performed using published scRNAseq datasets of whole decidual tissues from first-^25^ and third-^51^ trimester pregnancies as interacting cells with the dNK2, 3 and 4 subsets reported here (Supplementary Fig. S6D). The strongest receptor-ligand interaction scores were between extra villous trophoblast (EVT) HLA-G and dNK KLRD1, KLRC1, KIR3DL1, and KIR2DL4, which was overall consistent across first- and third- trimester EVT and dNK subsets (Supplementary Fig. S6D). However, unique to the dNK4 subset was the HLA-II-LAG-3 interaction, given the higher *LAG-3* expression in the dNK4 subset (Fig. 3J). The HLA-II-LAG-3 interactions were identified between dNK4 and decidual T cells and macrophages (dM) from first-trimester decidua, and hofbauer cells (HB), decidual T cells, macrophages and stromal cells from the third-trimester (Fig. 3J). We then analysed interactions with CD6 as our scRNAseq and flow cytometry data showed that CD6 was associated with the dNK4 subset. Receptor-ligand interaction analysis showed most interactions with CD6 occurred between dNK3 and dNK4 subsets with interacting cells from the third-trimester decidua (Fig. 3K). Additionally, the CXCL14-CXCR4 interaction was predominantly observed between dNK subsets and first-trimester decidual cell subsets, indicating a dynamic change in this interaction between early and late pregnancy (Fig. 3L).

### Pregnancy biases NK cells towards *HLA-D*, *AREG,* and *SOCS1* expression

To identify altered transcriptomic states of NK cells in pregnant peripheral blood, a pseudo-bulk differential gene expression analysis was performed between pregnant and non-pregnant groups. This identified a total of 361 differentially expressed genes (DEGs), with 194 genes upregulated in pregnant NK cells (56 genes with >2-fold increase) and 167 genes upregulated in non-pregnant NK cells (41 genes with >2-fold increase) (Fig. 4A; Supplementary Table S17). Remarkably, 7 of the 56 genes (12.5%) that had >2-fold increase in pregnant NK cells encoded HLA-II related genes (members of the HLA-D gene family and *CD74*) (Fig. 4A and B). We confirmed upregulation of HLA-II associated genes at the protein level, with increased expression of HLA-DR on pregnant peripheral blood and decidual NK cells (Fig. 4C). When HLA-II expression levels were assessed across clusters, the highest expression was on peripheral adaptive-like NK3 cells (Fig. 4D). We then performed specific analysis of CD56^dim^/NK1 cells (clusters 0 and 7) and CD56^bright^/NK2 cells between pregnant and non-pregnant women (Fig. 4E and F). Indeed, even within the CD56^dim^/NK1 subset, but not the CD56^bright^/NK2 subset, *HLA-DRB5* remained more highly expressed in pregnancy, indicating an association of increased HLA-II with pregnancy itself (Fig. 4E and F; Supplementary Fig. S7A; Supplementary Tables S18 and S19). In addition to upregulation of HLA-DR, we also observed higher expression of proteins associated with lymphocyte regulation including CD25/IL-2RA, CD39, CD73, CCR5, and PD-1 on pregnant blood and decidual NK cells, while CX3CR1, KLRG1, and CD158 (KIR) were increased on non-pregnant peripheral blood NK cells (Fig. 4C).

**Fig. 4:**
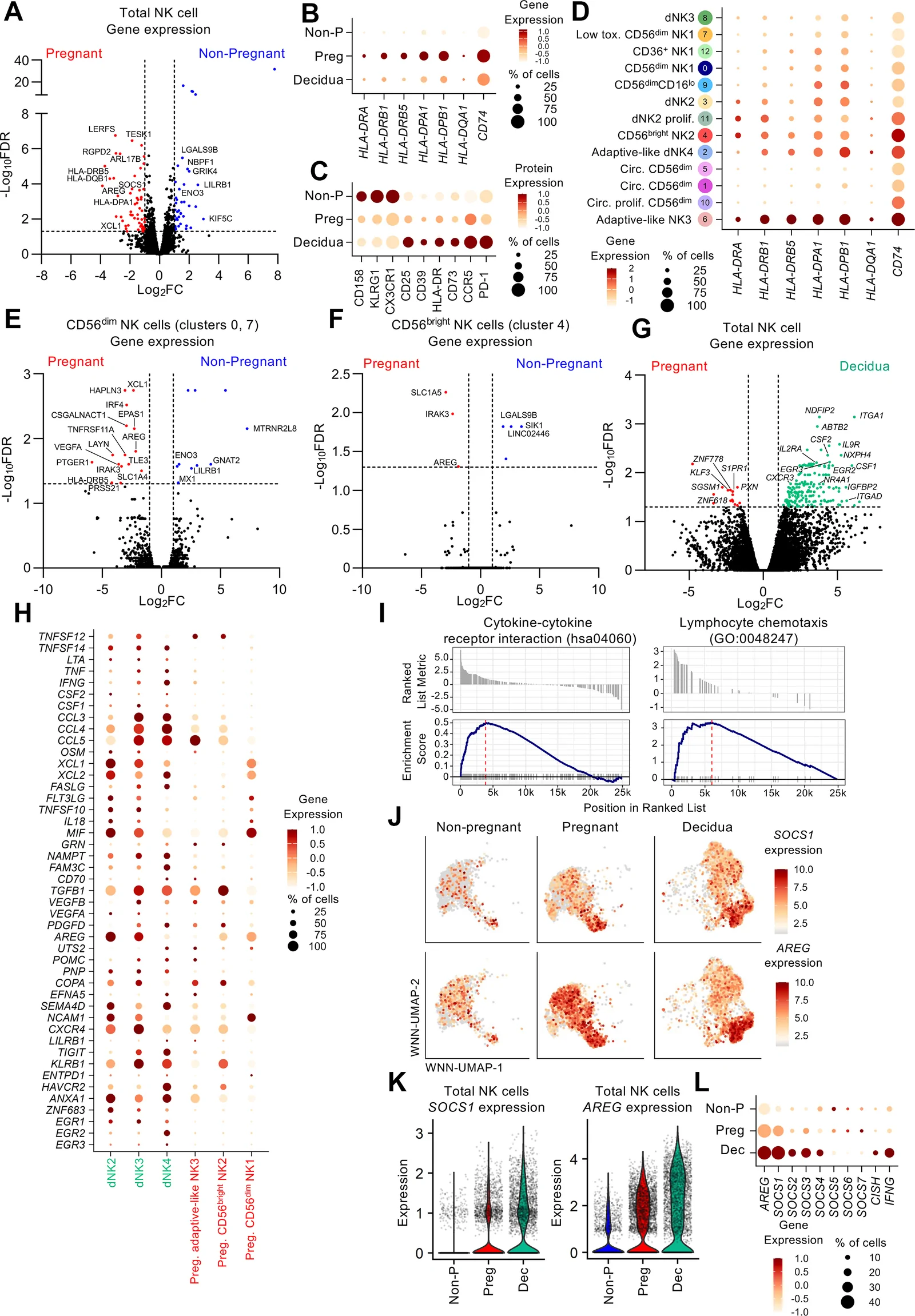
**Altered NK cell transcriptome and proteome during pregnancy.** Each panel contains data from pregnant (n = 3), non-pregnant (n = 2) peripheral blood or decidua (n = 3) samples. (A) Volcano plot of differential gene expression in NK cells between pregnant (n = 3) and non-pregnant (n = 2) groups. (B and C) Bubble plot of expression level and proportion of cells expressing selected HLA-II associated genes (B) and selected differentially expressed proteins (C) per group. (D) Bubble plot of expression level and proportion of cells expressing selected HLA-II genes per WNN-UMAP cluster. (E and F) Volcano plots of differential gene expression between pregnant (n = 3) and non-pregnant (n = 2) (E) CD56^dim^ and (F) CD56^bright^ NK cells. (G) Volcano plot of differential gene expression between pregnant peripheral NK (n = 3) and dNK (n = 3) cells. (H) Bubble plot of selected genes encoding receptors and ligands on dNK and peripheral blood NK subsets. (I) Gene set enrichment analyses of top KEGG (left) and GO (right) terms enriched in dNK based on normalised enrichment scores and gene set size. (J) WNN-UMAPs split by group of *SOCS1* (top) or *AREG* (bottom) expression. (K) Violin plots showing *SOCS1* or *AREG* expression per group. (L) Bubble plot depicting *AREG*, *SOCS1-7, CISH* and *IFNG* expression level and proportion of cells expressing per group. (B–D,J) Colour gradient depicts the gene/protein expression level. Circle size depicts the frequency of cells expressing. (A, E–G) Horizontal dashed lines depict statistical significance threshold, vertical dashed lines indicate the log_2_FC cutoff.

As described above, dNK cells have been defined in terms of their subsets within tissues, but comparisons to circulating NK cells from term pregnancies are lacking. Our analyses comparing peripheral blood NK cells from pregnancy to dNK cells revealed that dNK cells upregulated genes encoding cytokines/chemokines or soluble mediators, and their receptors including *CSF1, CSF2, XCL1, XCL2, AREG, CCL3, CCL4, CCL5,* and *CXCR4* (Fig. 4G and H, Supplementary Table S20). Aside from the lack of dNK1 in third-trimester decidua, there were many similarities in gene expression profiles between dNK subsets in our dataset and previous descriptions of dNK in first-trimester pregnancy.^25^ Similar to previous observations of dNK from first-trimester decidua, our third-trimester dNK2 subset maintained their characteristic increased expression of *XCL1, XCL2, MIF, ANXA1, w*hile dNK3 maintained higher *CCL3, CCL4, CCL5,* and *TGFB1* in comparison to dNK2 (Fig. 4H). The adaptive-like dNK4 subset was similar to dNK3 cells with high expression of *CCL3, CCL4, CCL5, TGFB1, PDGFD*, however in contrast to dNK2 and dNK3, had low expression of *AREG, CXCR4,* and *MIF* (Fig. 4H). dNK4 cells had the highest expression of exhaustion markers *TIGIT* and *HAVCR2* (encoding TIM-3), and soluble mediators *FASLG* and *IFNG*, indicating a higher level of activation in this subset (Fig. 4H). Supporting enhanced production and reactivity to cytokines in dNK more broadly, gene set enrichment analysis (GSEA) identified that in comparison to peripheral blood NK cells during pregnancy, dNK cells had enrichment for cytokine-cytokine receptor interaction (KEGG term hsa04060) and lymphocyte chemotaxis (GO term 0048247) gene sets, demonstrating enhanced capacity to secrete and respond to cytokines and soluble mediators (Fig. 4I). In addition to enhanced expression of cytokines, chemokines, and their receptors, dNK cells uniquely expressed transcription factors linked to tissue-residency (*ZNF683,* also known as *HOBIT*), and NK cell anergy (*EGR1*, *EGR2*, and *EGR3*) which are likely involved in modulating the altered gene profiles observed in comparison to the blood (Fig. 4G and H).

In addition to HLA-II associated genes, other key genes of interest upregulated in pregnancy were *SOCS1* (suppressor of cytokine signalling 1) and *AREG* due to their involvement in regulating immune responses (Fig. 4A). In fact, *AREG* was consistently upregulated across CD56^dim^/NK1 (clusters 0, 7) and CD56^bright^/NK2 (cluster 4) subsets in pregnancy, indicating a global increase in expression across all NK cells, although to a lesser magnitude in the CD56^bright^/NK2 subset (Fig. 4J and K; Supplementary Fig. S7B; Supplementary Tables S18 and S19). Given *AREG* is a defining feature of peripheral blood CD56^bright^/NK2 cells in a healthy state,^47^ we reveal that higher *AREG* expression is observed in CD56^dim^/NK1 cells during pregnancy. Furthermore, expression of these key genes of interest (*SOCS1* and *AREG*) in decidual NK cells as compared to pregnant peripheral blood was overall similar, with modestly higher expression of *SOCS1* (log_2_FC = 0.599) and *AREG* (log_2_FC = 0.197) in dNK cells, predominantly in the dNK2 (cluster 3) and dNK3 (cluster 8) subsets (Fig. 4G,J,K; Supplementary Table S20).

Suppressor of cytokine signalling (SOCS) proteins constitute a network of inhibitory molecules that regulate immune cell activation. We analysed expression levels of SOCS family member genes *SOCS1-7*, *CISH*, and cytokines *IFNG* to determine a pattern of expression for these related molecules. *SOCS1* had the highest expression level across all SOCS genes, especially in pregnant peripheral and dNK cells, while expression of other SOCS family member genes was relatively low (Fig. 4L). Compared to pregnant blood, dNK cells had higher expression of *CISH*, increased expression levels of *SOCS2, SOCS3* and *SOCS4*, decreased *SOCS6* and *SOCS7* (Fig. 4G, L; Supplementary Table S20). While amphiregulin and SOCS1 do not directly interact with each other, they both have direct interactions with KIT, EGFR, and IGF1R, which are linked to JAK/STAT signalling pathways (Supplementary Fig. S7C). Using publicly available datasets of first- and third-trimester circulating and dNK cells, we observed increased expression of *SOCS1* and *AREG* in the third-trimester dNK cells. *AREG* expression was more highly expressed in dNK cells derived from the decidual parietalis than basalis (within Garcia’s dataset) in the third-trimester (Supplementary Fig. S7D). This suggests that pregnancy increases expression of regulatory molecules *AREG* and *SOCS1*, potentially as a mechanism to tightly control NK cell activation.

### Integrating multiomics to reveal NK cells phenotypic shifts in early and late pregnancy

Our high dimensional datasets revealed key differentially expressed genes and proteins in NK cells during pregnancy. We integrated these findings to define the kinetics of selected immune marker expression (CD6, CCR5, CD9, CD36, CX3CR1, CD39, CD73, HLA-DR, and amphiregulin (Areg)) in circulating NK cells in early and late pregnancy by staining fresh whole blood from first-trimester and term third-trimester pregnancies, and non-pregnant individuals (Supplementary Fig. S8A). In comparison to non-pregnant women, pregnant women in the first-trimester had reduced frequencies of NK cells expressing CX3CR1, while both first and third-trimester pregnancy had reduced CD9 and CD36, and CD6 remained similar (Fig. 5A–D). We observed that CD9 and CD36 were highly co-expressed, and there were lower frequencies of CD9^+^CD36^+^ NK cells in both first- and third-trimester pregnancy (Fig. 5D). There were no differences in frequencies of CD39^+^ NK cells, while there was a small reduction in CD73^+^ NK cells frequencies between first- and third-trimester pregnancy, although only a minor subset of NK cells expressed these markers (Supplementary Fig. S8B and C). In contrast to the higher *AREG* expression observed in our scRNAseq dataset, pregnant women in the third-trimester had lower frequencies of Areg^+^ NK cells (Supplementary Fig. S8D and E). Meanwhile, in concordance with our scRNAseq dataset, HLA-DR and CCR5 were both expressed at an increased frequency in first and third-trimester pregnancy, indicating that these changes occur early and are maintained throughout pregnancy (Fig. 5E–H).

**Fig. 5:**
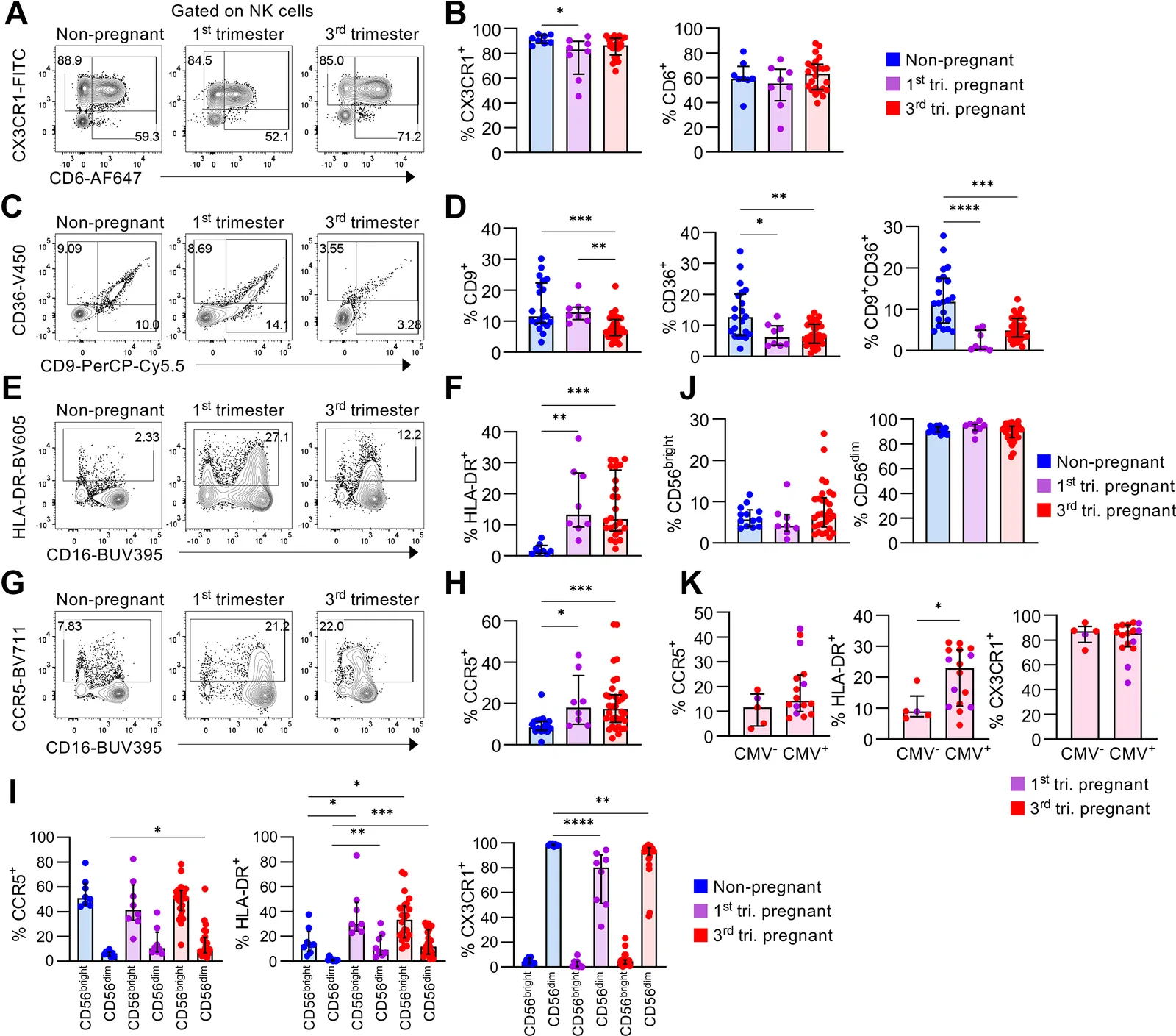
**NK cell phenotype dynamics in early and late pregnancy.** (A and B) Representative FACS plots (A) and frequencies of CX3CR1^+^ and CD6^+^ (B) NK cells in non-pregnant (n = 8), first-trimester pregnant (n = 8), and third-trimester pregnant (n = 24). (C and D) Representative FACS plots (C) and frequencies of CD9^+^ and CD36^+^ (D) NK cells in non-pregnant (n = 21), first-trimester pregnant (n = 8), and third-trimester pregnant (n = 36). (E and F) Representative FACS plots (E) and frequencies of HLA-DR^+^ (F) NK cells in non-pregnant (n = 8), first-trimester pregnant (n = 8), and third-trimester pregnant (n = 24) blood. (G and H) Representative FACS plots (G) and frequencies of CCR5^+^ (H) NK cells in non-pregnant (n = 20), first-trimester pregnant (n = 8), and third-trimester pregnant (n = 24) blood. (I) Frequencies of CCR5^+^, HLA-DR^+^, and CX3CR1^+^ NK cells within the CD56^bright^ and CD56^dim^ subsets in non-pregnant (n = 8), first-trimester pregnant (n = 8), and third-trimester pregnant (n = 24) blood. A Kruskal–Wallis test was used to compare groups for each of the CD56^bright^ and CD56^dim^ populations. (J) Frequencies of CD56^bright^ and CD56^dim^ NK cells in non-pregnant (n = 13), first-trimester pregnant (n = 8), and third-trimester pregnant (n = 15) blood. (K) Frequencies of CCR5^+^, HLA-DR^+^, and CX3CR1^+^ NK cells between CMV sero-negative (CMV^−^; n = 5) and sero-positive (CMV^+^; n = 16). A Mann–Whitney U test was used to determine statistical significance. (B,D,F,H) A Kruskal–Wallis test was used to determine statistical significance. (B,D,F,H-K) p-values denoted as: ∗p < 0.05, ∗∗p < 0.01, ∗∗∗p < 0.001, ∗∗∗∗p < 0.0001. Medians and IQR are shown.

Given the roles of CX3CR1, HLA-DR, and CCR5 in cell migration and activation, we further assessed their expression on the CD56^bright^ and CD56^dim^ subsets. CCR5 and HLA-DR expression was associated with CD56^bright^ subset, while CX3CR1 was associated with the CD56^dim^ subset (Fig. 5I).

We next compared expression of CCR5, HLA-DR, and CX3CR1 between non-pregnant and pregnant groups’ CD56^bright^ and CD56^dim^ subsets. While CCR5 was more highly expressed in the CD56^bright^ subset, there was significantly higher expression in the CD56^dim^ subset in third-trimester pregnancy as compared to non-pregnant women (Fig. 5I). HLA-DR had increased frequencies in both the CD56^bright^ and CD56^dim^ subsets for both first- and third-trimester pregnancies, while CX3CR1 was only reduced in first- and third-trimester pregnancies within the CD56^dim^ subset (Fig. 5I).

To understand whether these phenotypic differences were a result of skewing in the proportions of the CD56^bright^ and CD56^dim^ subsets we compared their frequencies across pregnancy groups and observed no differences (Fig. 5J). Furthermore, knowing that CMV infection history can impact on NK cell phenotypes, we compared frequencies of CCR5, HLA-DR, and CX3CR1 expressing NK cells in CMV^+^ and CMV^−^ pregnancies (Fig. 5K). While CCR5 and CX3CR1 were not affected by CMV status, there was significantly higher frequencies of HLA-DR^+^ NK cells in CMV^+^ pregnancies (Fig. 5K).

### SOCS1 expression linked with cytokine production and stimulation

Given the role of SOCS1 in limiting cellular production of IFN-γ,^52^ we further probed gene expression in *SOCS1*^+^ and *SOCS1*^−^ NK cell subsets in the blood and decidua to gain phenotypic insight. Genes associated with the CD56^bright^ subset, including *IL7R, COTL1, XCL1, XCL2, AREG, CD3E, TCF7,* and *CD44,* had higher expression in *SOCS1*^+^ NK cells from the blood (Fig. 6A; Supplementary Table S21). Like the peripheral blood subset, *SOCS1*^+^ dNK cells had increased levels of *AREG* and *XCL1* in addition to *ZFP36L2, CAPG, KLRC1, TNFRSF18, ZNF683 (HOBIT), IGFBP2,* and *CD52* (Fig. 6B; Supplementary Table S22). Knowing that CD56^bright^ NK cells are “cytokine producers”, increased expression of *SOCS1* in this subset would potentially reduce NK cell cytokine production and responsiveness to infections. Importantly, pregnant peripheral blood and dNK cells had higher proportions of *SOCS1*^+^ NK cells (31.8% and 60.6%, respectively) than in non-pregnant blood (13.9%), suggesting that pregnancy may induce NK cells that are primed for rapid SOCS1 expression following exposure to cytokines (Fig. 6C). Between the blood and decidua, there were four additional shared genes in *SOCS1*^+^ NK cells, namely *XCL1, CAPG, AREG* and *KLRC1* (Fig. 6D and E). This gene signature was also enriched in *SOCS1*^−^ dNK, indicating the decidual microenvironment might play a role in upregulating these genes.

**Fig. 6:**
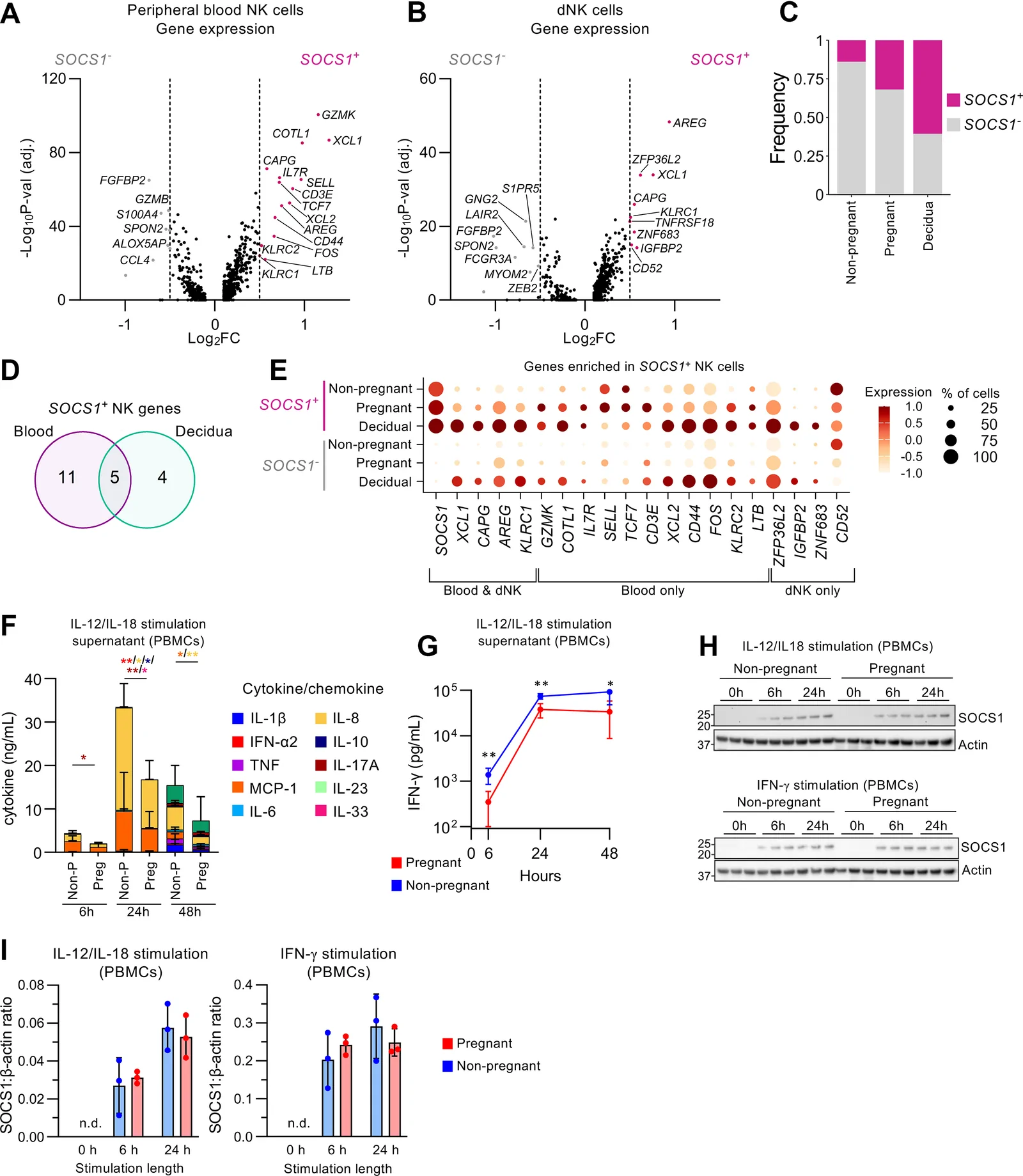
**SOCS1 expression linked to cytokine production and stimulation.** (A and B) Volcano plots of differential gene expression between *SOCS1*^+^ and *SOCS1*^−^ NK cells from (A) peripheral blood (pregnant (n = 3), non-pregnant (n = 2)) and (B) decidua (n = 3; excluding adaptive-like NK cells). Horizontal dashed lines depict statistical significance threshold, vertical dashed lines indicate the log_2_FC cutoff. (C) Frequency of *SOCS1*^+^ and *SOCS1*^−^ NK cells in non-pregnant (n = 2) and pregnant (n = 3) peripheral blood, and decidua (n = 3). (D) Venn diagram of genes expressed in *SOCS1*^+^ NK cells from peripheral blood and decidua. (E) Bubble plot of expression level and proportion of cells expressing genes upregulated in *SOCS1*^+^ NK cells across *SOCS1*^+^ and *SOCS1*^−^ NK cells in non-pregnant (n = 2) and pregnant (n = 3) peripheral blood, and decidua (n = 3). (F) Cytokine concentrations in supernatants after pregnant (n = 5) or non-pregnant (n = 5) PBMC stimulation with IL-12/IL-18 for 6, 24, or 48 h. Mean and SDs are shown. (G) IFN-γ concentration in supernatants after IL-12/IL-18 stimulation of PBMCs from pregnant (n = 5) or non-pregnant (n = 5) individuals. (H) Immunoblotting of SOCS1 and β-actin after 0, 6, or 24 h stimulation with IL-12/IL-18 or IFN-γ in pregnant (n = 3) and non-pregnant (n = 3) PBMCs. (I) Densitometry of SOCS1 expression relative to actin after IL-12/IL-18 or IFN-γ stimulation of pregnant (n = 3) and non-pregnant (n = 3) samples. Mean and SD are shown. (F, G, I) Statistical significance determined with Mann–Whitney U tests. p-values denoted as: ∗p < 0.05, ∗∗p < 0.01, ∗∗∗p < 0.001, ∗∗∗∗p < 0.0001.

SOCS1 is known to be induced by IFN-γ and acts in a negative feedback loop to limit further IFN-γ signalling.^53^ To probe the functional consequence of increased *SOCS1* during pregnancy, we used IL-12/IL-18 stimulation on PBMCs, which is known to induce potent IFN-γ production.^54^^,^^55^ Indeed, we observed reduced secretion of multiple cytokines, including IFN-α, IL-8, IL-10, IL17A, and IL-33 and IFN-γ, in supernatants after IL-12/IL-18 stimulation at 6, 24 and 48 h in samples from pregnancy (Fig. 6F and G). To link this with SOCS1 expression, we next investigated protein SOCS1 levels by immunoblotting to understand whether SOCS1 protein expression was also increased in pregnancy (Fig. 6H). Directly ex vivo, SOCS1 protein was not detectable in either pregnant or non-pregnant samples but was robustly induced in response to IL-12/IL-18 and IFN-γ in both groups (Fig. 6H and I). Given that in pregnancy there was enhanced control of IFN-γ secretion, but SOCS1 protein induction remained similar between pregnant and non-pregnant women, future studies into the exact significance of higher *SOCS1* transcription in NK cells during pregnancy are needed.

## Discussion

In this study, we identified key molecules involved in the altered immunological state of pregnancy using high-throughput cell surface proteome screening of >350 markers and scRNAseq with 130 CITE-seq antibodies. We identified a unique population of CD57^+^ dNK4 cells with adaptive-like features, which can be distinguished from the dNK1-3 subsets, and that there are dynamic changes to the proportions of dNK subsets across first- and third-trimester pregnancy. Additionally, while many existing studies focus on understanding immunity at the maternal–foetal interface (i.e., decidua), we aimed to fill an important gap in our understanding of the impact pregnancy has on circulating NK cells, as this could have implications in regard to respiratory viral infection severity. We identified at a phenotypic level higher frequencies of NK cells expressing CCR5 and HLA-DR, and reduced frequencies of NK cells expressing CX3CR1, CD9, and CD36 in the blood using cross-sectional samples from early and late pregnancy. At the transcriptomic level, we revealed higher expression of regulatory molecules *AREG* and *SOCS1*, and HLA-D genes in circulating NK cells from third-trimester pregnancy.

Higher HLA-II expression in NK cells from pregnant women was striking, given it was observed at both RNA and protein levels, and in early and late pregnancy. This is especially interesting given the role of HLA-II on NK cells is not well defined or understood, therefore this finding provides insights into mechanisms of NK cell function. HLA-II molecules are known for their role in antigen presentation but are also described as activation markers for T and NK cells.^2^^,^^29^ HLA-DR expression is also associated with adaptive-like or “trained” NK cells,^56^ often described as NKG2C^+^/CD57^+^, and have been shown to increase with multigravidity.^57^ The increased frequencies of HLA-DR^+^ NK cells in the blood during pregnancy could be indicative of expansions of trained NK cells. From a functional perspective, little is known about HLA-II^+^ NK cells, however in a mouse model MHC-II^+^ NK cells resulted in reduced antigen-specific CD4^+^ T cell activation due to competitive antigen presentation between MHC-II^+^ NK cells, which lack costimulatory molecules CD80/CD86, and professional antigen-presenting dendritic cells.^58^ While trogocytosis is one mechanism for NK cells to acquire HLA-II, which has been shown in CD8^+^ T cells,^59^ our findings show increased HLA-II at both transcriptional and protein levels, suggesting an intrinsic upregulation of HLA-II. Frequencies of HLA-DR^+^ NK cells are often increased in response to viral infections like COVID-19^60^ and decrease upon recovery. Importantly, our previous work showed the expected increase in HLA-DR^+^ NK cells during COVID-19 does not occur in pregnancy because baseline frequencies are already increased.^29^ In the present study, we corroborate this further by showing that NK cells from pregnant women have higher expression of HLA-II RNA and that higher frequencies of HLA-DR^+^ NK cells are already present in first-trimester pregnancy, indicating this is an early adaptation. Further studies are required to understand the biological significance of this, but it could be a potential regulatory mechanism that controls HLA-II-dependent immune cell activation in the context of pregnancy and viral infections.

Migratory markers CX3CR1 and CCR5 were differentially expressed between NK cells from pregnant and non-pregnant women. We identified a strong association of CX3CR1 with CD56^dim^ cells and CCR5 with CD56^bright^ cells. CX3CR1 has been shown to play an important role in NK cell adhesion and ability to lyse target cells,^61^, ^62^, ^63^ and its expression allows for migration towards CX3CL1.^64^ Given that CX3CR1 expression was decreased in CD56^dim^ NK cells from the first- and third-trimester pregnant women, this could potentially be due to increased migration towards CX3CL1, which is highly expressed in the decidua.^65^ In contrast, CCR5 was increased on NK cells from pregnant women. While the role of CCR5 on NK cells during pregnancy is not well-defined, NK cell migration to the lung during influenza A virus infection is partially CCR5-dependent.^66^, ^67^, ^68^ Therefore, an increase in CCR5^+^ NK cells during pregnancy could enhance NK cell migration to the lung during respiratory virus infections and offer protection, or alternatively, contribute to hyperactivation and disease severity.

We identified differential expression of molecules involved in cell adhesion, including CD9 and CD36. Co-expression of CD9 and CD36 has been described on monocytes/macrophages^69^ and platelets,^70^ while CD9 is known to be expressed on dNK cells.^71^ Co-expression of CD9 and CD36 is not well described on peripheral blood NK cells. Our analyses showed that ∼15% of peripheral blood NK cells co-expressed CD9 and CD36, with a lower frequency (∼8%) observed during pregnancy. A potential mechanism underpinning the presence of CD9 and CD36 on NK cells, and their reduced frequency during pregnancy is platelet conjugation. A study investigating immunity to COVID-19 found ICU patients had an increase in NK cell-platelet aggregates, based on the identification of cells expressing NK cell markers CD56 and CD16, and platelet markers CD62P and CD41.^72^ There is a known increase in platelet-platelet aggregation during pregnancy,^73^^,^^74^ which could affect the number of platelets available for NK cell binding, resulting in the observed reduced frequency of CD9^+^CD36^+^ NK cells in pregnant women.

Expression of *SOCS1* and *AREG* were increased at the transcriptional level in NK cells from pregnant women. SOCS1 is a member of the SOCS family (CIS, SOCS1-7), which are induced by inflammation and have key roles in regulating immune responses.^75^, ^76^, ^77^ SOCS1 is primarily known for its ability to inhibit JAK-STAT signalling in response to type I (IFNα/β) and type II (IFN-γ) interferons to control inflammation.^77^ Importantly, previous literature suggests SOCS1 is critical in regulating inflammation, as *socs1*^−/−^ mice experience neonatal fatality due to uncontrolled IFN-γ production, which could be treated through administration of neutralising anti-IFN-γ antibodies.^78^, ^79^, ^80^ This could be an important potential mechanism relevant to pregnancy complications, as prior studies suggest pregnancy complications can be linked to conditions with excess type I IFN, which triggers SOCS1 production, such as in systemic lupus erythematosus.^81^^,^^82^ Amphiregulin is also known for having a regulatory role in immune responses, as it is strongly associated with CD4^+^ regulatory T cells and type II immunity.^83^^,^^84^ Interestingly, we observed that *AREG* expression was highest in dNK cells. Others have shown that AREG ^+^ dNK cells are implicated in decidualization.^85^ When we investigated whether SOCS1 and amphiregulin shared downstream signalling pathways and we identified that both molecules are involved in JAK-STAT signal transduction, suggesting there may be altered signalling during pregnancy. Indeed, our in vitro stimulation data demonstrated reduced production of IFN-γ in pregnancy, providing mechanistic insight to explain the dampening of NK cell inflammatory potential, which is likely beneficial to pregnancy, but may negatively impact anti-viral responses.

Our analysis of dNK cells in early and late pregnancy identified the CD39^+^ dNK1 subset is at higher frequencies in the first trimester, and essentially absent at the end of the third pregnancy trimester. This is in support of prior studies that show the presence of dNK1 in early pregnancy,^25^ and provides new insight on dNK cell dynamics throughout pregnancy. One mechanism underlying a decrease in dNK1 could be reduced NK migration from the blood to the decidua, as previous study analysing NK subsets in uterus transplant patients showed that all CD39^+^KIR^+^ NK cells in the recipient were recipient-derived, suggesting they are replenished from the periphery.^71^ Additionally, previous studies have shown higher expression of *GZMA*, *GZMB*, *GNLY*, and *PRF1* in dNK1,^25^ and higher dNK1 frequencies in the first trimester.^86^ The higher frequencies of dNK1 during the first trimester reported here could reflect a need for increased cytotoxic activity during spiral artery remodelling and decidualisation in the first trimester. This suggests that later in pregnancy, there is less infiltration of NK cells to the decidua compared to early pregnancy, perhaps because the vasculature is already well established.

Importantly, we revealed a subset of adaptive-like dNK4 cells that share similar gene signatures to that of the circulating NK3 subset, while also expressing tissue-residency marker CD49a. Adaptive-like NK cells are known to undergo expansions in people with CMV, however a direct link between adaptive-like NK cells and protection from CMV symptoms or severity has not been established. CMV in pregnancy is of great concern, with 42% of pregnancies in CMV^+^ mothers experiencing a non-primary CMV infection,^50^ and primary CMV infection in pregnancy resulting in vertical transmission rates of 30% in the first trimester and 72% in the third trimester.^87^ Importantly, congenital CMV infection is linked to birth defects, including permanent vision impairments caused by chorioretinitis, hearing loss, cerebral palsy, seizures, and in rare cases, death.^88^^,^^89^ Here, we report that adaptive-like dNK4 cells have increased expression of adhesion molecule CD6, exhaustion molecule TIM-3, in addition to cytotoxic granzymes and perforin. Importantly, previous studies have shown that CMV infection can upregulate the endogenous granzyme B inhibitor, SERPINB9.^90^ This makes the increased expression of *GZMH* and *PRF1* in dNK4 a potential valuable alternative mechanism to control CMV infections within the placenta and prevent vertical transmission.

In summary, our high-dimensional datasets generated from this study provide key knowledge on the immunological landscape of pregnancy. Our study used high-dimensional approaches to explore phenotypic and transcriptional differences in NK cell immunity in third-trimester pregnancy. While we validated our findings in larger sample sizes of different individuals at both first and third trimesters and identified similar patterns in existing datasets, the interpretation of the initial screening analysis is limited by the relatively small sample size due to sample availability, experiment feasibility, and costs associated with the multiomic approaches. The exploratory nature of the study and limited data on the key genes and proteins of interest on NK cells and in pregnancy meant that we could not estimate an effect size for a power calculation. Additionally, while the ages between the pregnant and non-pregnant groups was not significantly different, the median age was different between groups. Due to a limited sample size, we could not account for confounders such as age, CMV status, gravidity and parity, and we did not have complete medical history information. Furthermore, mechanical and enzymatic digestion of decidual tissue may alter gene and protein expression profile and may not directly reflect the tissue in vivo. Nonetheless, it is important to consider the physiological state of pregnancy in understanding immunity towards infections and cancers, as the needs of immune cells often differ to non-pregnant individuals, yet pregnant women are often excluded from clinical trials due to safety concerns. Therefore, data provided here can potentially inform vaccine and therapeutic strategies, specifically geared towards managing infections, diseases and complications of pregnancy.

## Contributors

KK led the study. KK and LCR supervised the study. JRH, THON, LCR and KK designed the experiments. JRH, LCR, THON, LFA, MJS, LK, EKA, RRH, KD, XJ, AAM, MVP, AIH, NLM, PMS, CD, TK, and SN performed and analysed experiments. JRH, SL, IT, JS and JCC analysed data. PMS, AC, ME, CX, H-FK, MAAKK, LKM, ADB, SEN, AGB and PGT provided crucial reagents. NdA, KB, DAW, LH, ML, SW and NJH recruited or provided patient cohorts. JRH, THON, LCR, and KK wrote the manuscript. JRH, SL, LCR and KK have accessed and verified the underlying study data. All authors read and approved the final version of the manuscript.

## Data sharing statement

No data are missing or excluded from the study. Raw sequencing data have been made available via the Gene Expression Omnibus (https://www.ncbi.nlm.nih.gov/geo/) with accession number: GSE302014, LegendScreen data can be viewed via an interactive shiny app (https://habel-nk.shinyapps.io/dashboard/), and all other data is available as source data (Supplementary Material).

## Declaration of interests

PGT consulted for Sanofi, Merck, Pfizer, JNJ, Shennon Bio, Immunoscape and RA Ventures, received financial support from Sanofi, holds stock or stock options in Shennon Bio, and received funding from the National Institutes of Allergy and Infectious Diseases (NIAID) and National Institutes of Health (NIH) under contract nos. RO1AI136514, 75N93019C00052, 75N93021C0016, AI170116, and AI165077. KK received honoraria from Pfizer and AstraZeneca. All other authors declare no competing interests.

## Appendix A Supplementary data

Source_Data

Supplemental_Western_Blots
