# Supplementary material for: Multidimensional molecular profiling reveals decidual and circulating NK cell dynamics in early and late pregnancy
Source: eBioMedicine. 2026 Oct 1;132:106495. doi: 10.1016/j.ebiom.2026.106495 (PMC13657211; doi:10.1016/j.ebiom.2026.106495)
Supplement: Supplementary file 1 [file mmc1.pdf]

Multidimensional molecular profiling reveals decidual and circulating NK cell dynamics in early and late pregnancy

Jennifer R Habel[1], Thi H O Nguyen[1], Lilith F Allen[1], Morgan J Skinner[1], Lukasz Kedzierski[1], E Kaitlynn Allen[2], Shihan Li[3], Natasha de Alwis[4,5], Ruth R Hagen[1], Karen Doggett[6,7], Xiaoxiao Jia[1], Ilariya Tarasova[3], Anastasia A Minervina[2,8], Mikhail V Pogorelyy[2,8], Ashleigh I Holloway[1], Nikita L. Mathew[1], Philippa M Saunders[1], Allison Clatch[1], Maximilien Evrard[1], Calvin Xu[1], Hui-Fern Koay[1], Md Abdullah-Al-Kamran Khan[1], Laura K Mackay[1], Alexander D Barrow[1], Celia Douros[9], Theo Karapanagiotidis[9], Suellen Nicholson[9], Katherine Bond[9-11], Deborah A Williamson[9,11-13], Lisa Hui[4,5,14,15], Martha Lappas[4], Susan Walker[4,5], Sandra E Nicholson[6,7], Natalie J Hannan[4,5], Andrew G Brooks[1], Jan Schroeder[3], Jeremy Chase Crawford[16], Paul G Thomas[2,8], Louise C Rowntree[1]* and Katherine Kedzierska[1]*
*authors contributed equally

1. Department of Microbiology and Immunology, University of Melbourne, Peter Doherty Institute for Infection and Immunity, Melbourne, Victoria, Australia
2. Department of Immunology, St. Jude Children's Research Hospital, Memphis, Tennessee, USA
3. Computational Sciences Initiative, Department of Immunology and Microbiology, University of Melbourne at the Peter Doherty Institute for Infection and Immunity, Melbourne, Australia
4. Department of Obstetrics, Gynaecology and Newborn Health, University of Melbourne, Heidelberg, Victoria, Australia
5. Mercy Perinatal, Mercy Hospital for Women, Heidelberg, Victoria, Australia
6. The Walter and Eliza Hall Institute of Medical Research, Melbourne, Victoria, Australia
7. Department of Medical Biology, University of Melbourne, Melbourne, Victoria, Australia
8. Immunology and Vaccine Development Program, Vaccine and Infectious Disease Division, Fred Hutchinson Cancer Center, Seattle, Washington, USA
9. Victorian Infectious Diseases Reference Laboratory at the Peter Doherty Institute for Infection and Immunity, Melbourne, Victoria, Australia
10. Department of Microbiology, Royal Melbourne Hospital, Melbourne, Victoria, Australia
11. Victorian Infectious Diseases Service, Royal Melbourne Hospital, at the Peter Doherty Institute for Infection and Immunity, Melbourne, Victoria, Australia
12. Department of Infectious Diseases, The University of Melbourne at the Peter Doherty Institute for Infection and Immunity, Melbourne, Victoria, Australia
13. School of Medicine, University of St Andrews, St Andrews, SCT, UK
14. Northern Health, Epping, Victoria, Australia
15. Reproductive Epidemiology Group, Murdoch Children's Research Institute, Parkville, Victoria, Australia
16. Department of Host-Microbe Interactions, St. Jude Children's Research Hospital, Memphis, Tennessee, USA

Correspondence:
kkedz@unimelb.edu.au

Supplementary Table 1. Cohort Table.

Supplementary Table 2. Age and Gestation.

Supplementary Table 3. NK cell marker panel 1

Specificity	Fluorophore	Dilution	Catalog number	RRID
LiveDead	Aqua	1:800	L34957 (Thermo Fisher)	Not applicable
HLA-DR	BV605	1:50	307640 (Biolegend)	AB_2561913
TIM-3	BV650	1:100	565564 (BD)	AB_2722547
CD19	BV711	1:100	563036 (BD)	AB_2737968
CD56	BV786	1:100	564058 (BD)	AB_2738569
CD36	APC	1:50	336208 (Biolegend)	AB_1279224
CD16	AF700	1:50	302026 (Biolegend)	AB_2278418
CD14	APC-H7	1:50	560180 (BD)	AB_1645464
CD9	PE	1:100	312106 (Biolegend)	AB_2075892
CD3	PE-CF594	1:800	562280 (BD)	AB_11153674
TIGIT	PE-Cy7	1:100	372714 (Biolegend)	AB_2632929

Supplementary Table 4. NK cell marker panel 2

Specificity	Fluorophore	Dilution	Catalog number	RRID
CD16	BUV395	1:50	563785 (BD)	AB_2744293
CD73	BUV737	1:50	612812 (BD)	AB_2870137
CD36	V450	1:50	561535 (BD)	AB_10893025
NKp46	BV510	1:50	331924 (Biolegend)	AB_2563854
HLA-DR	BV605	1:50	307640 (Biolegend)	AB_2561913
CD39	BV650	1:50	563681 (BD)	AB_2738370
CCR5	BV711	1:50	359129 (Biolegend)	AB_2734387
CD56	BV786	1:50	564058 (BD)	AB_2738569
CD6	AF647	1:25	564260 (BD)	AB_2868516
NKG2A	AF700	1:50	375120 (Biolegend)	AB_2888868
CD14	APC-H7	1:50	560180 (BD)	AB_1645464
CD19	APC-H7	1:50	560177 (BD)	AB_1645470
LiveDead	NIR	1:50	L34976 (Thermo Fisher)	Not applicable
CX3CR1	FITC	1:50	341606 (Biolegend)	AB_1626272
CD9	PerCP-Cy5.5	1:50	561329 (BD)	AB_2869566
CD74	PE	1:50	326808 (Biolegend)	AB_2075505
CD3	PE-CF594	1:800	562280 (BD)	AB_11153674
Areg	PE-Cy7	1:50	25-5370-42 (Thermo Fisher)	AB_2716952

Supplementary Table 5. Decidual NK cell subset panel

Specificity	Fluorophore	Dilution	Catalog number	RRID
TIM-3	BUV737	1:100	568680 (BD)	AB_3684463
CD45	BUV805	1:200	612892 (BD)	AB_2870180
HLA-DR	BV605	1:100	307640 (Biolegend)	AB_2561913
CD39	BV650	1:100	563681 (BD)	AB_2738370
CD56	BV786	1:50	564058 (BD)	AB_2738569
CD6	AF647	1:50	564260 (BD)	AB_2868516
CD14	APC-H7	1:50	560180 (BD)	AB_1645464
CD19	APC-H7	1:50	560177 (BD)	AB_1645470
LiveDead	NIR	1:800	L34976 (Thermo Fisher)	Not applicable
CD103	FITC	1:100	984806 (Biolegend)	AB_2936603
CD9	PerCP-Cy5.5	1:100	561329 (BD)	AB_10613815
CD49a	PE	1:50	328304 (Biolegend)	AB_1236407
CD3	PE-CF594	1:800	562280 (BD)	AB_3703462
CD52	PE-Cy7	1:100	316012 (Biolegend)	AB_2650809

Supplementary Table 6. LEGENDScreen PE mAbs. Provided as Excel table.

Supplementary Table 7. Donor barcoding for LEGENDScreen

Sample	Fluorophore 1	Fluorophore 2	Fluorophore 3
Dec1	BUV395		
Dec2	BUV496		
Dec3	BUV805		
Dec4	BUV395	BUV496	
Preg1	BUV395	BUV805	
Preg2	BUV496	BUV805	
Preg3	BUV395	CTV	
Preg4	BUV496	CTV	
NonP1	BUV805	CTV	
NonP2	BUV395	BUV496	BUV805
NonP3	BUV395	BUV496	CTV
NonP4	BUV395	BUV805	CTV

Supplementary Table 8. LEGENDScreen backbone panel

Antigen	Fluorophore	Dilution	Catalog number	RRID
CD45-biotin*	BUV395-SAV	1:100 (SAV)	564176 (BD)	AB_2869553 (SAV)
LiveDead	Blue	1:500	L34961 (ThermoFisher)	Not applicable
CD45-biotin*	BUV496-SAV	1:200 (SAV)	612961 (BD)	AB_2870237 (SAV)
CD25	BUV563	1:50	612918 (BD)	AB_2870203
CD95	BUV615	1:100	752346 (BD)	AB_2875863
PD-1	BUV661	1:100	750260 (BD)	AB_2874457
γδTCR	BUV737	1:100	748533 (BD)	AB_2872944
CD45-biotin*	BUV805-SAV	1:100 (SAV)	564923 (BD)	AB_2869629 (SAV)
MR1-5-OP-RU tetramer	BV421-SAV	1:100	405225 (Biolegend)	Not applicable
CTV	450-violet	1:2000	562158 (BD)	Not applicable
CD103	BV480	1:50	746472 (BD)	AB_2743774
CD45RA	BV510	1:50	304142 (Biolegend)	AB_2561947
CD19	BV570	1:200	302236 (Biolegend)	AB_2563606
CD161	BV605	1:50	339916 (Biolegend)	AB_2563607
CD4	BV650	1:200	563875 (BD)	AB_2744425
CD27	BV711	1:200	563167 (BD)	AB_2738042
HLA-DR	BV750	1:100	307672 (Biolegend)	AB_2800802
CD38	BV786	1:100	563964 (BD)	AB_2738515
CD56	BB515	1:25	564488 (BD)	AB_2744428
CD14	PerCP	1:100	325632 (Biolegend)	AB_2563328
CD8	PerCP-Cy5.5	1:100	565310 (BD)	AB_2687497
LEGENDScreen mAb	PE	1:100	700011 (Biolegend)	AB_3097146
CD3	PE-CF594	1:800	562280 (BD)	AB_3703462
CD1d-α-GalCer tetramer	PE-Cy7	1:100	405206 (Biolegend)	Not applicable
TCR-Vα7.2	APC	1:50	351708 (Biolegend)	AB_10933246
CD16	AF700	1:50	302026 (Biolegend)	AB_2278418
CD69	APC-Cy7	1:50	310914 (Biolegend)	AB_314849

*CD45-biotin catalog number: 304004 (Biolegend) RRID: AB_314392

Supplementary Table 9. Fluorescent antibody panel to bulk sort prior to scRNAseq

Specificity	Fluorophore	Dilution	Catalog number	RRID
CD56	BB515	1:10	564488 (BD)	AB_2744428
CD8	PerCP-Cy5.5	1:100	565310 (BD)	AB_2687497
CD3	PacBlue	1:100	300418 (Biolegend)	AB_493095
LiveDead	Ghost Dye 510	1:150	13-0870-T100 (Cytek)	Not applicable
CD14	BV510	1:100	301842 (Biolegend)	AB_2561946
Streptavidin	BV510	1:600	563261 (BD)	Not applicable
IgD	PE-Cy7	1:500	561314 (BD)	AB_10642457
CD19	BV711	1:100	563036 (BD)	AB_2737968

Supplementary Table 10. LEGENDScreen differentially expressed proteins between pregnant versus non-pregnant blood groups. Provided as Excel table.

Supplementary Table 11. LEGENDScreen differentially expressed proteins between pregnant blood versus decidua basalis. Provided as Excel table.

Supplementary Table 12. LEGENDScreen differentially expressed proteins between cord blood versus pregnant blood. Provided as Excel table.

Supplementary Table 13. LEGENDScreen differentially expressed proteins between cord blood versus decidua basalis. Provided as Excel table.

Supplementary Table 14. scRNAseq/CITE-seq UMAP cluster descriptions

Cluster	Definition	Description	Reference
Cluster 0	Cytotoxic $CD56^{dim}CD16^{+}$	Conventional $CD56^{dim}CD16^{+}$ peripheral NK cells with high expression of *GZMA, GZMB, GNLY* and *PRF1*.	Rebuffet et al.
Cluster 1	Circulation-derived $CD56^{dim}$	Low/no CD49a or CD69 expression, likely spillover from blood into decidual tissue. Excluded from decidual analyses.	n.a.
Cluster 2	CMV-induced adaptive-like dNK	Adaptive-like dNK cells with characteristics of CMV-induced phenotype.	Lopez-Vergès, et al. Rebuffet et al.
Cluster 3	dNK2-like	Lack of CD39/*ENTPD1* and CD103/*ITGAE*, and presence of CD11c/*ITGAX* and *KLRB1*.	Vento-Tormo, et al.
Cluster 4	$CD56^{bright}CD16^{-}$	Conventional $CD56^{bright}CD16^{-}$ NK cells with low expression of *GZMA, GZMB* and *PRF1*.	Rebuffet et al.
Cluster 5	Circulation-derived $CD56^{dim}$	Low/no CD49a or CD69 expression, likely spillover from blood into decidual tissue. Excluded from decidual analyses.	Vento-Tormo, et al.
Cluster 6	CMV-induced adaptive-like	Adaptive-like NK cells with characteristics of CMV-induced phenotype.	Lopez-Vergès, et al. Rebuffet et al.
Cluster 7	Low cytotoxic $CD56^{dim}CD16^{+}$	Conventional $CD56^{dim}CD16^{+}$ NK cells with lower expression of *GZMA, GZMB, GNLY* and *PRF1*.	n.a.
Cluster 8	dNK3	$CD56^{bright}CD16^{-}$ with CD103/*ITGAE* and *KLRB1* expression.	Vento-Tormo, et al.
Cluster 9	$CD56^{dim}CD16^{lo}$	$CD56^{dim}$ subset with lower expression of CD16	n.a.
Cluster 10	Circulation-derived proliferating $CD56^{dim}$	Increased expression of tubulin genes *TUBB* and *TUBA1B* indicates proliferation. Low/no CD49a or CD69 expression, likely spillover from blood into decidual tissue. Excluded from decidual analyses.	n.a.
Cluster 11	dNK2-like proliferating	Lack of CD39/*ENTPD1* and CD103/*ITGAE*, and presence of CD11c/*ITGAX* and *KLRB1*, with increased expression of tubulin genes *TUBB* and *TUBA1B* indicates proliferation.	Vento-Tormo, et al.
Cluster 12	$CD36^{+}$ $CD56^{dim}CD16^{+}$	Conventional $CD56^{dim}CD16^{+}$ NK cells with high expression of *GZMA, GZMB, GNLY* and *PRF1* and additional expression of CD36.	n.a.
Cluster 13	Undefined (<25 cells)	n.d.	n.a.

n.a.: not applicable; n.d.: not determined

Supplementary Table 15. Differentially expressed genes between UMAP cluster 2 versus UMAP clusters 3 and 8. Provided as Excel table.

Supplementary Table 16. Differentially expressed genes between UMAP clusters 0 and 7 versus UMAP cluster 6. Provided as Excel table.

Supplementary Table 17. Differentially expressed genes in total NK cells between non-pregnant versus pregnant groups. Provided as Excel table.

Supplementary Table 18. Differentially expressed genes in $CD56^{dim}$ NK cells between non-pregnant versus pregnant groups. Provided as Excel table.

Supplementary Table 19. Differentially expressed genes in $CD56^{bright}$ NK cells between non-pregnant versus pregnant groups. Provided as Excel table.

Supplementary Table 20. Differentially expressed genes in total NK cells between decidua basalis versus pregnant blood groups. Provided as Excel table.

Supplementary Table 21. Differentially expressed genes between *SOCS1*$^{+}$ versus *SOCS1*$^{-}$ NK cells from pregnant/non-pregnant blood groups. Provided as Excel table.

Supplementary Table 22. Differentially expressed genes between *SOCS1*$^{+}$ versus *SOCS1*$^{-}$ NK cells from decidua basalis. Provided as Excel table.

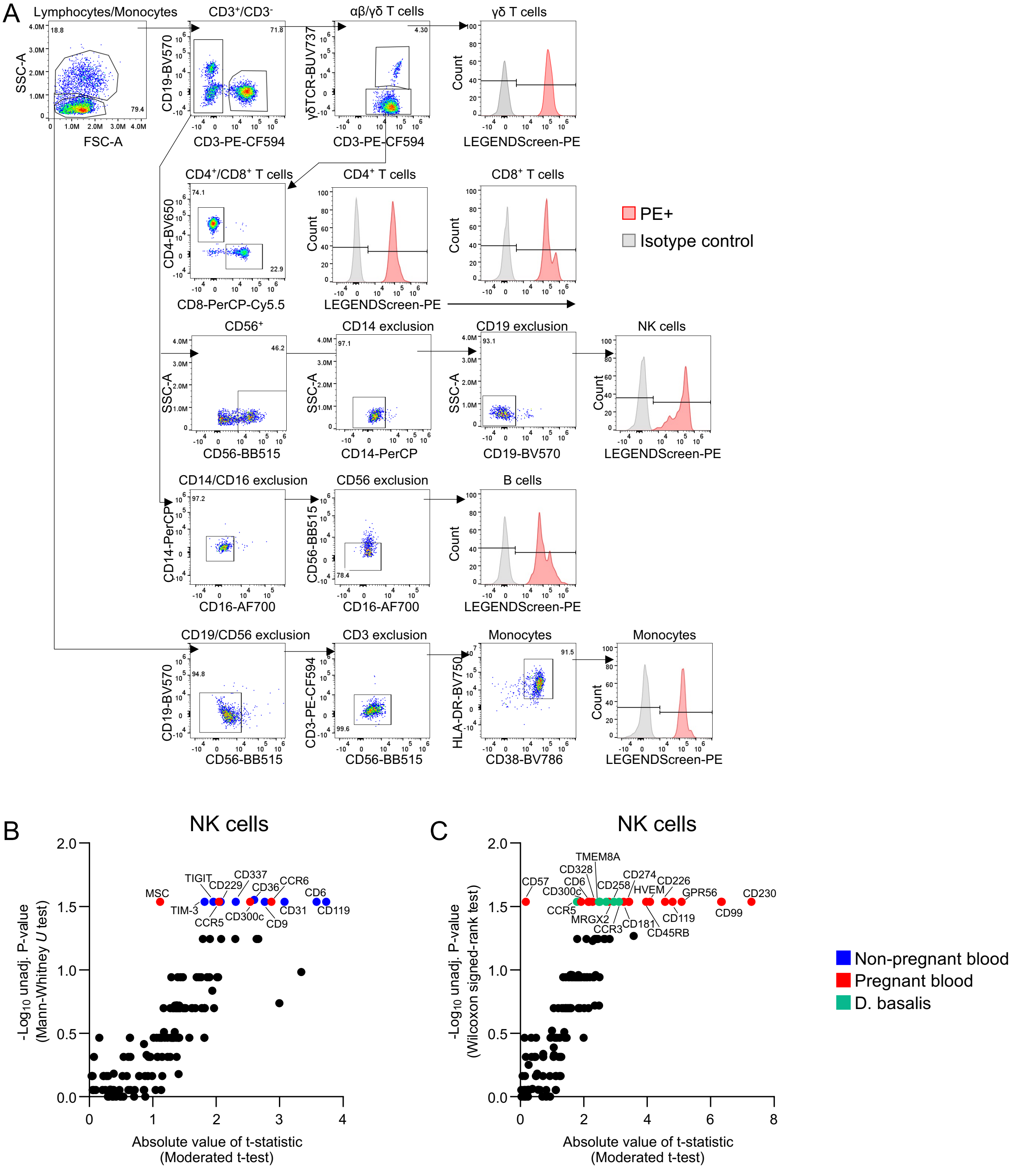
A
Lymphocytes/Monocytes
SSC-A
FSC-A
CD3+/CD3-
CD19-BV570
CD3-PE-CF594
αβ/γδ T cells
γδTCR-BUV737
γδ T cells
Count
LEGENDScreen-PE
CD4+/CD8+ T cells
CD4-BV650
CD8-PerCP-Cy5.5
CD4+ T cells
CD8+ T cells
PE+
Isotype control
CD56+
CD56-BB515
CD14 exclusion
CD14-PerCP
CD19 exclusion
CD19-BV570
NK cells
CD14/CD16 exclusion
CD16-AF700
CD56 exclusion
B cells
CD19/CD56 exclusion
CD3 exclusion
Monocytes
HLA-DR-BV750
CD38-BV786
B
NK cells
-Log10 unadj. P-value (Mann-Whitney U test)
Absolute value of t-statistic (Moderated t-test)
MSC
TIGIT
TIM-3
CD229
CCR5
CD337
CD300c
CD36
CCR6
CD9
CD31
CD6
CD119
C
NK cells
-Log10 unadj. P-value (Wilcoxon signed-rank test)
Absolute value of t-statistic (Moderated t-test)
CD57
TMEM8A
CD328
CD6
CD300c
CCR5
MRGX2
CD258
CCR3
CD274
HVEM
CD181
CD45RB
CD226
CD119
GPR56
CD99
CD230
Non-pregnant blood
Pregnant blood
D. basalis

Supplementary Figure 1. (A) Gating strategy from one representative sample to identify NK cells, monocytes, B cells, $CD8^+$ T cells, $CD4^+$ T cells, and γδ T cells. Gating of LEGENDScreen mAb-PE signals (red) on histograms was based on respective isotype control (grey). (B) Correlation between the uncorrected Mann-Whitney U test p-value and the absolute value of the t-statistic from a moderated t-test for differential LEGENDScreen protein analysis in circulating NK cells from pregnant and non-pregnant women. (C) Correlation between the uncorrected Wilcoxon signed-rank test p-value and the absolute value of the t-statistic from a moderated t-test for differential LEGENDScreen protein analysis in NK cells from third trimester pregnant blood and decidua.

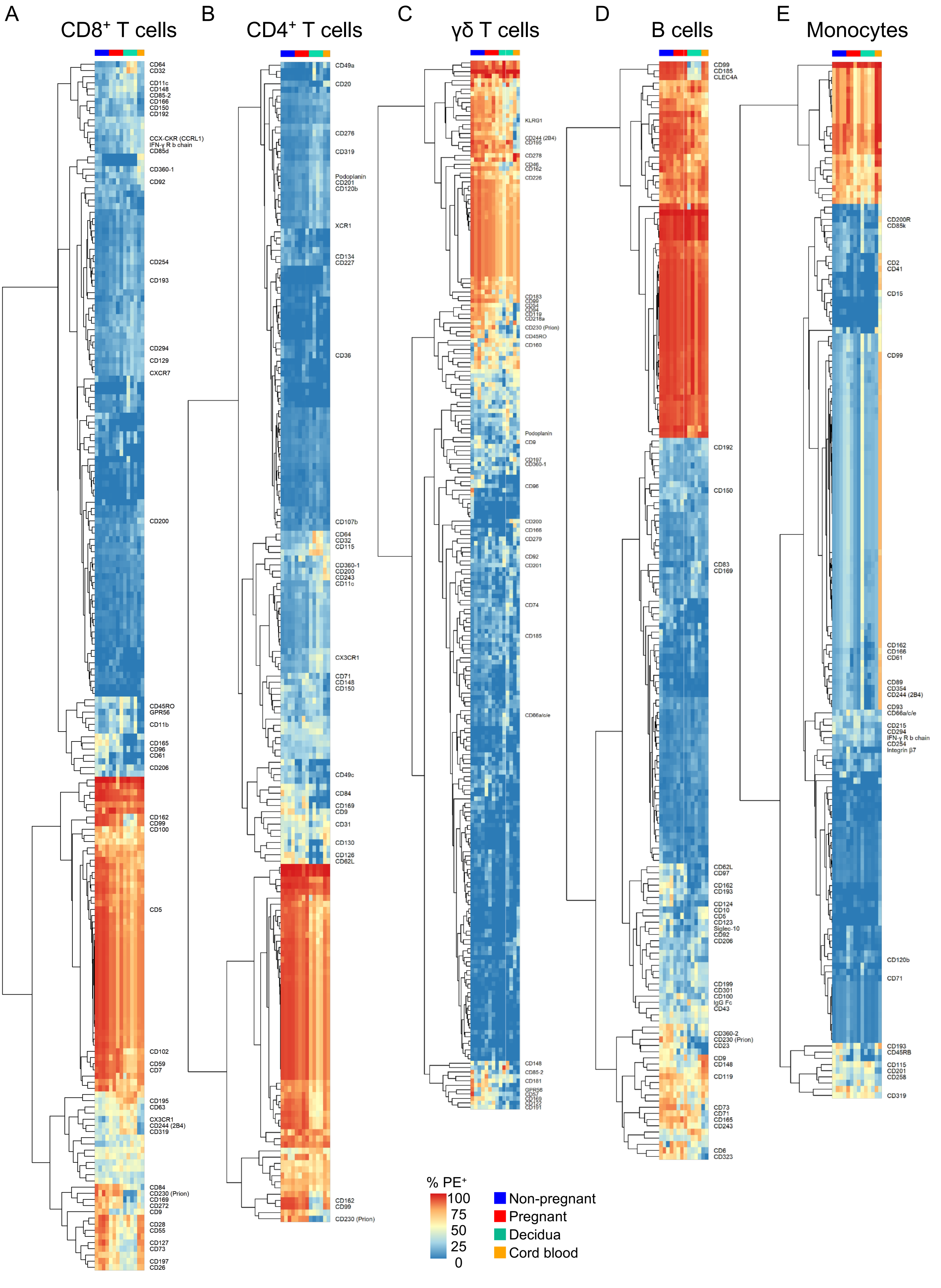
A
CD8+ T cells
CD64
CD32
CD11c
CD148
CD85-2
CD166
CD150
CD192
CCX-CKR (CCRL1)
IFN-γ R b chain
CD85d
CD360-1
CD92
CD254
CD193
CD294
CD129
CXCR7
CD200
CD45RO
GPR56
CD11b
CD165
CD96
CD61
CD206
CD162
CD99
CD100
CD5
CD102
CD59
CD7
CD195
CD63
CX3CR1
CD244 (2B4)
CD319
CD84
CD230 (Prion)
CD169
CD272
CD9
CD28
CD55
CD127
CD73
CD197
CD26
B
CD4+ T cells
CD49a
CD20
CD276
CD319
Podoplanin
CD201
CD120b
XCR1
CD134
CD227
CD36
CD107b
CD64
CD32
CD115
CD360-1
CD200
CD243
CD11c
CX3CR1
CD71
CD148
CD150
CD49c
CD84
CD169
CD9
CD31
CD130
CD126
CD62L
CD162
CD99
CD230 (Prion)
C
γδ T cells
KLRG1
CD244 (2B4)
CD195
CD278
CD46
CD162
CD226
CD183
CD99
CD54
CD94
CD119
CD218a
CD230 (Prion)
CD45RO
CD160
Podoplanin
CD9
CD197
CD360-1
CD96
CD200
CD166
CD279
CD92
CD201
CD74
CD185
CD66a/c/e
CD148
CD85-2
CD181
GPR56
CD57
CD169
CD272
CD191
D
B cells
CD99
CD185
CLEC4A
CD192
CD150
CD83
CD169
CD62L
CD97
CD162
CD193
CD124
CD10
CD5
CD123
Siglec-10
CD92
CD206
CD199
CD301
CD100
IgG Fc
CD43
CD360-2
CD230 (Prion)
CD23
CD9
CD148
CD119
CD73
CD71
CD165
CD243
CD6
CD323
E
Monocytes
CD200R
CD85k
CD2
CD41
CD15
CD99
CD162
CD166
CD61
CD89
CD354
CD244 (2B4)
CD93
CD66a/c/e
CD215
CD294
IFN-γ R b chain
CD254
Integrin β7
CD120b
CD71
CD193
CD45RB
CD115
CD201
CD258
CD319
% PE+
100
75
50
25
0
Non-pregnant
Pregnant
Decidua
Cord blood

Supplementary Figure 2. (A-E) Heatmaps with hierarchical clustering of columns showing frequencies of marker-PE expression on $CD8^{+}$ T cells (A), $CD4^{+}$ T cells (B), γδ T cells (C), B cells (D), and monocytes (E) from pregnant (n=4) and non-pregnant (n=4) peripheral blood, cord blood (n=2) and decidua (n=4). Markers expressed on >10% of cells in at least one sample were included for analysis (196 (A), 188 (B), 238 (C), 178 (D), and 168 (E) out of total 342 markers).

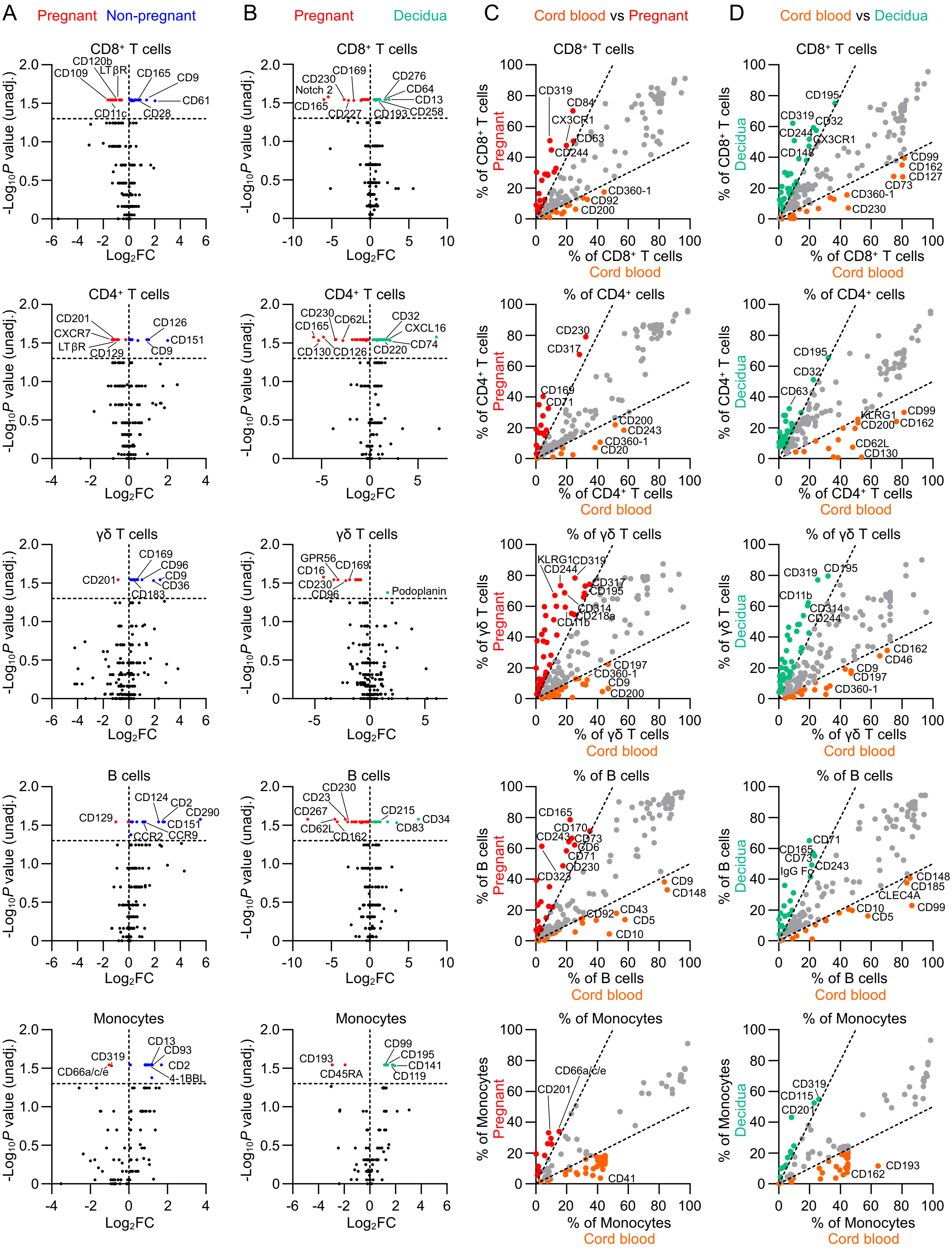

A Pregnant Non-pregnant
B Pregnant Decidua
C Cord blood vs Pregnant
D Cord blood vs Decidua
CD8⁺ T cells
CD4⁺ T cells
γδ T cells
B cells
Monocytes
-Log10P value (unadj.)
Log2FC
CD120b CD109 LTβR CD165 CD9 CD61 CD11c CD28
CD230 CD169 Notch 2 CD165 CD227 CD276 CD64 CD13 CD193 CD258
CD319 CD84 CX3CR1 CD63 CD244 CD360-1 CD92 CD200
CD195 CD319 CD32 CD244 CX3CR1 CD148 CD99 CD162 CD127 CD73 CD360-1 CD230
CD201 CXCR7 LTβR CD129 CD126 CD151 CD9
CD230 CD165 CD62L CD130 CD126 CD32 CXCL16 CD220 CD74
% of CD4⁺ cells
CD230 CD317 CD169 CD71 CD200 CD243 CD360-1 CD20
CD195 CD32 CD63 CD99 KLRG1 CD200 CD162 CD62L CD130
CD169 CD96 CD201 CD9 CD36 CD183
GPR56 CD169 CD16 CD230 CD96 Podoplanin
% of γδ T cells
KLRG1 CD319 CD244 CD317 CD195 CD314 CD218a CD11b CD197 CD360-1 CD9 CD200
CD319 CD195 CD11b CD314 CD244 CD162 CD46 CD9 CD197 CD360-1
CD124 CD2 CD290 CD129 CD151 CCR2 CCR9
CD230 CD23 CD267 CD62L CD162 CD215 CD34 CD83
% of B cells
CD165 CD170 CD243 CD73 CD6 CD71 CD230 CD323 CD9 CD148 CD92 CD43 CD5 CD10
CD71 CD165 CD73 CD243 IgG Fc CD148 CD185 CLEC4A CD99 CD10 CD5
CD13 CD93 CD319 CD2 CD66a/c/e 4-1BBL
CD99 CD195 CD193 CD141 CD45RA CD119
% of Monocytes
CD66a/c/e CD201 CD41
CD319 CD115 CD201 CD193 CD162
% of CD8⁺ T cells Pregnant
% of CD8⁺ T cells Decidua
% of CD8⁺ T cells Cord blood
% of CD4⁺ T cells Pregnant
% of CD4⁺ T cells Decidua
% of CD4⁺ T cells Cord blood
% of γδ T cells Pregnant
% of γδ T cells Decidua
% of γδ T cells Cord blood
% of B cells Pregnant
% of B cells Decidua
% of B cells Cord blood
% of Monocytes Pregnant
% of Monocytes Decidua
% of Monocytes Cord blood

Supplementary Figure 3. (A) Volcano plots depicting differential protein expression in non-pregnant (n=4) vs pregnant (n=4) peripheral blood immune cell populations including, $CD8^{+}$ T cells, $CD4^{+}$ T cells, γδ T cells, B cells and monocytes. (B) Volcano plots depicting differential protein expression in decidual (n=4) vs pregnant blood (n=4) immune cell populations. Volcano plot horizontal dashed line indicates the log_{10} p-value cutoff of 1·3 (p=0.05). (C,D) Scatter plot of average percent of cells expressing for (C) cord blood (n=2) vs pregnant blood (n=4) and (D) cord blood (n=2) vs decidual (n=4) $CD8^{+}$ T cells, $CD4^{+}$ T cells, γδ T cells, B cells, monocytes. Scatter plot dashed lines indicate a log_2FC cut-off of 1.

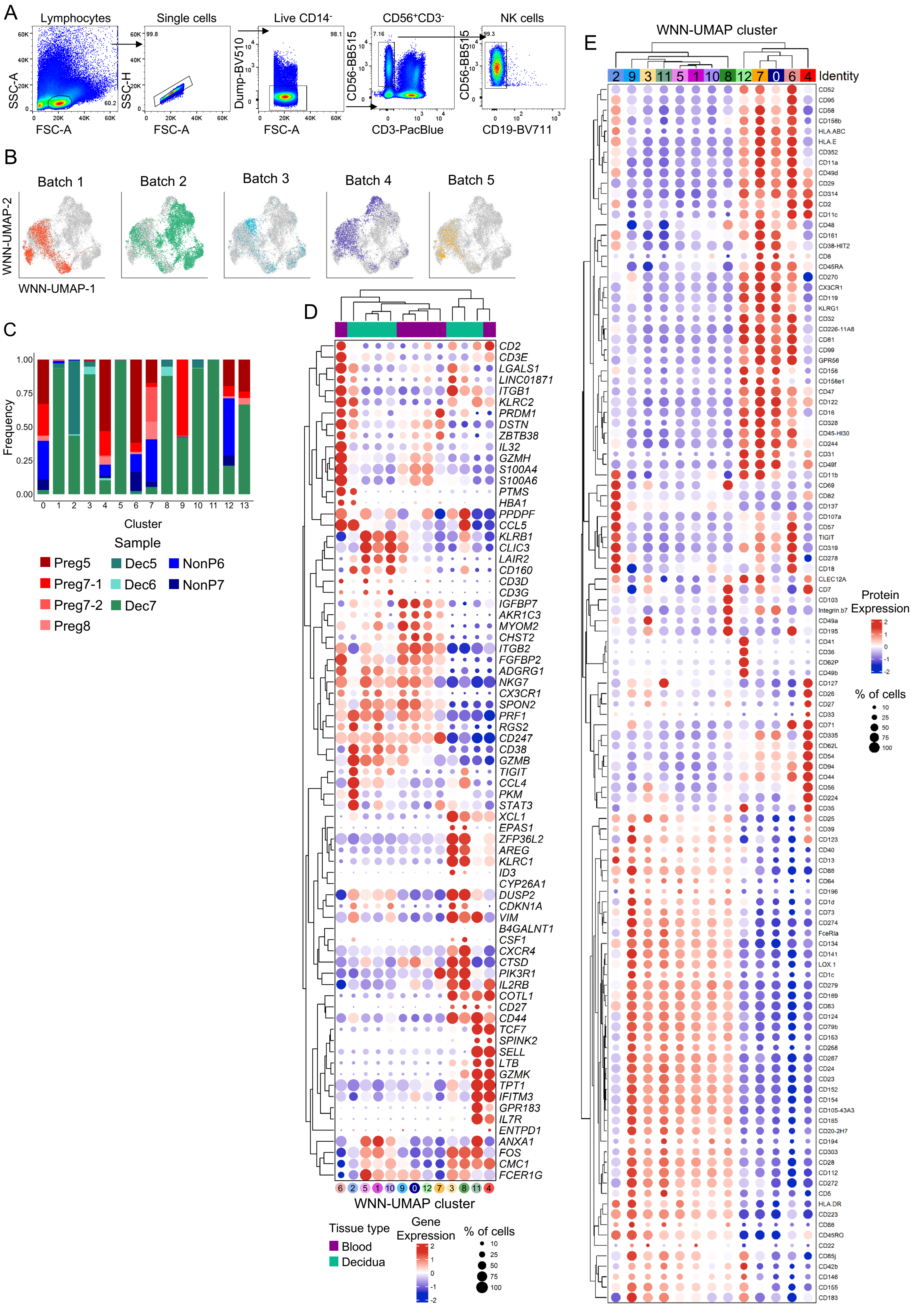

A
Lymphocytes
Single cells
Live CD14-
CD56+CD3-
NK cells
B
Batch 1
Batch 2
Batch 3
Batch 4
Batch 5
WNN-UMAP-2
WNN-UMAP-1
C
Frequency
Cluster
Sample
Preg5
Preg7-1
Preg7-2
Preg8
Dec5
Dec6
Dec7
NonP6
NonP7
D
WNN-UMAP cluster
Tissue type
Blood
Decidua
Gene Expression
% of cells
E
WNN-UMAP cluster
Identity
Protein Expression
% of cells

Supplementary Figure 4. Sort gating strategy and cluster features in single-cell RNA sequencing (A) Gating strategy for bulk NK cells. (B) WNN-UMAP coloured by 10x reaction batches. (C) Proportion of each sample (pregnant peripheral blood NK cells (reds), non-pregnant peripheral blood NK cells (blues), decidual NK cells (greens) per WNN-UMAP cluster. (D) Hierarchically clustered dot plot of 79 genes associated with NK cell subsets across each WNN-UMAP cluster. (E) Hierarchically clustered dot plot of CITE-seq protein markers across each WNN-UMAP cluster.

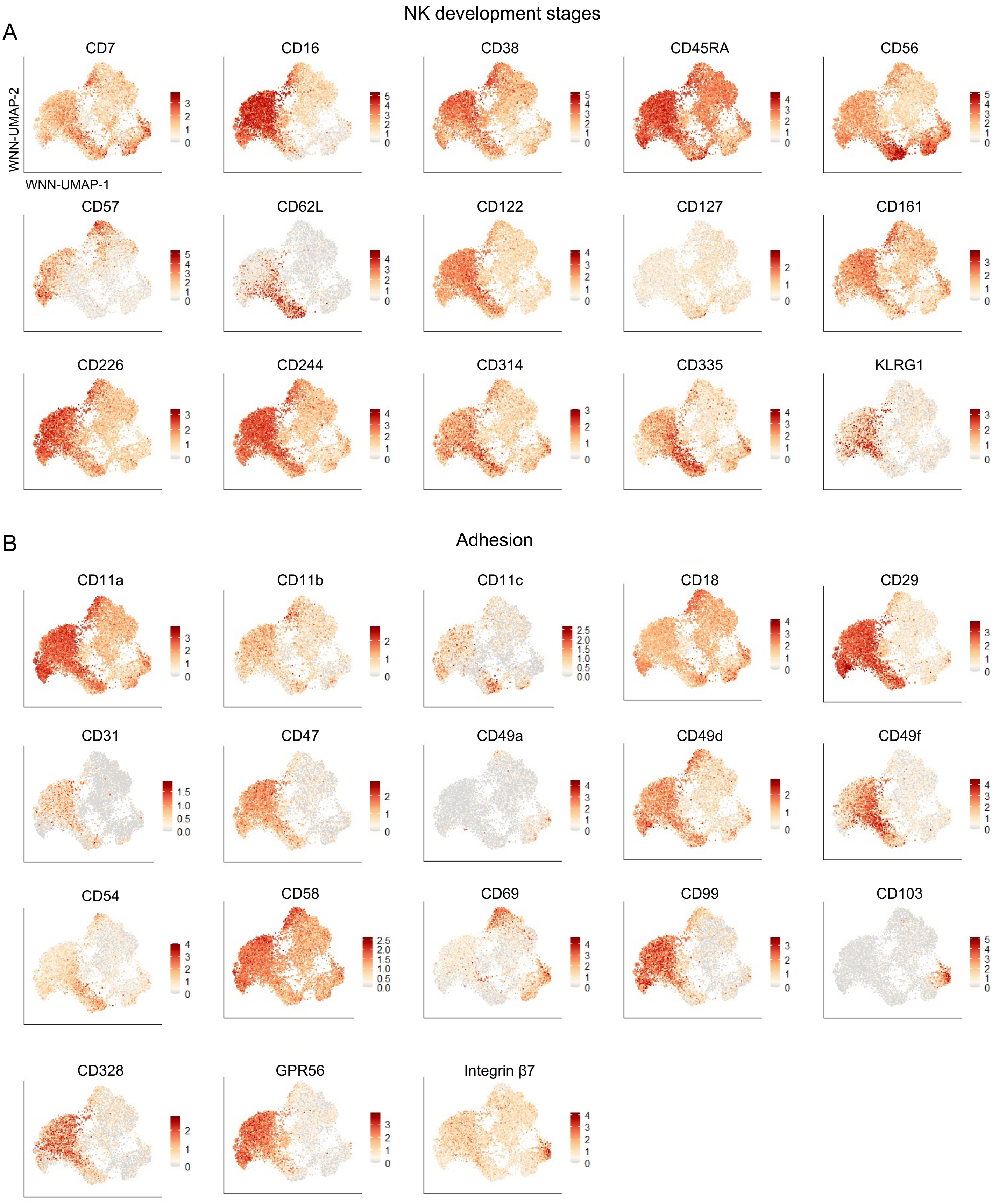
A
NK development stages
CD7
CD16
CD38
CD45RA
CD56
WNN-UMAP-2
WNN-UMAP-1
CD57
CD62L
CD122
CD127
CD161
CD226
CD244
CD314
CD335
KLRG1
B
Adhesion
CD11a
CD11b
CD11c
CD18
CD29
CD31
CD47
CD49a
CD49d
CD49f
CD54
CD58
CD69
CD99
CD103
CD328
GPR56
Integrin β7

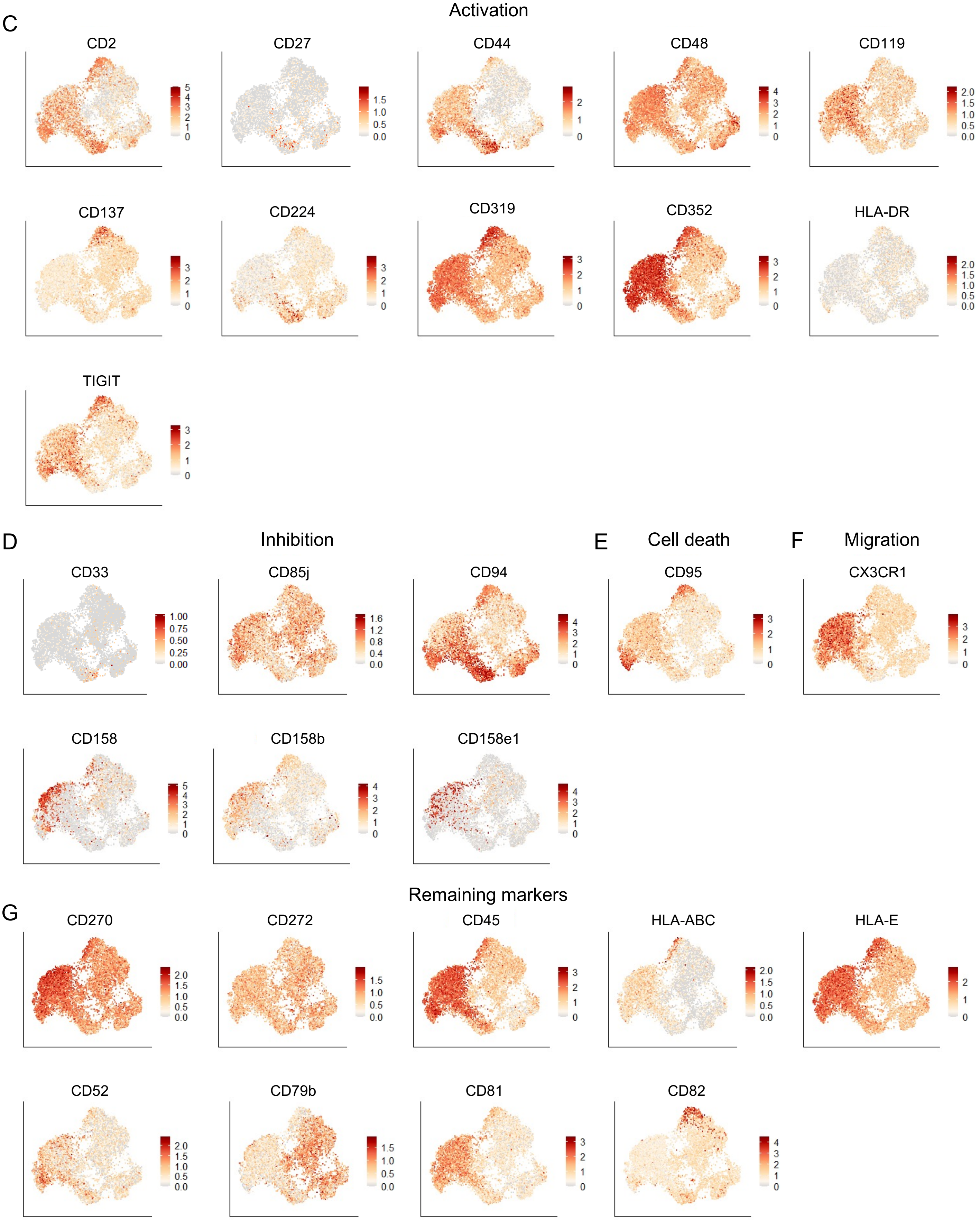
C
Activation
CD2
CD27
CD44
CD48
CD119
CD137
CD224
CD319
CD352
HLA-DR
TIGIT
D
Inhibition
CD33
CD85j
CD94
CD158
CD158b
CD158e1
E
Cell death
CD95
F
Migration
CX3CR1
G
Remaining markers
CD270
CD272
CD45
HLA-ABC
HLA-E
CD52
CD79b
CD81
CD82

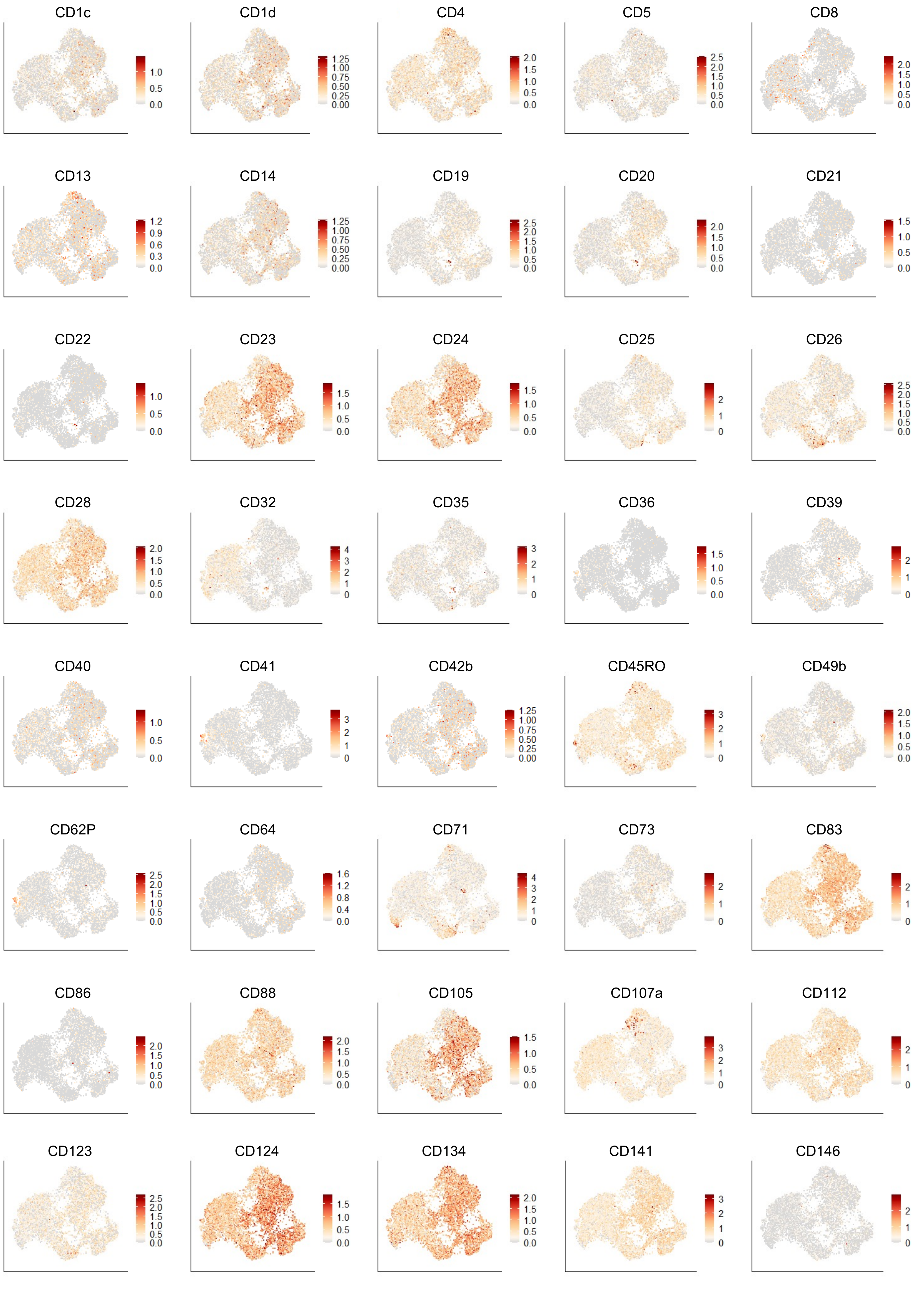
CD1c
CD1d
CD4
CD5
CD8
CD13
CD14
CD19
CD20
CD21
CD22
CD23
CD24
CD25
CD26
CD28
CD32
CD35
CD36
CD39
CD40
CD41
CD42b
CD45RO
CD49b
CD62P
CD64
CD71
CD73
CD83
CD86
CD88
CD105
CD107a
CD112
CD123
CD124
CD134
CD141
CD146

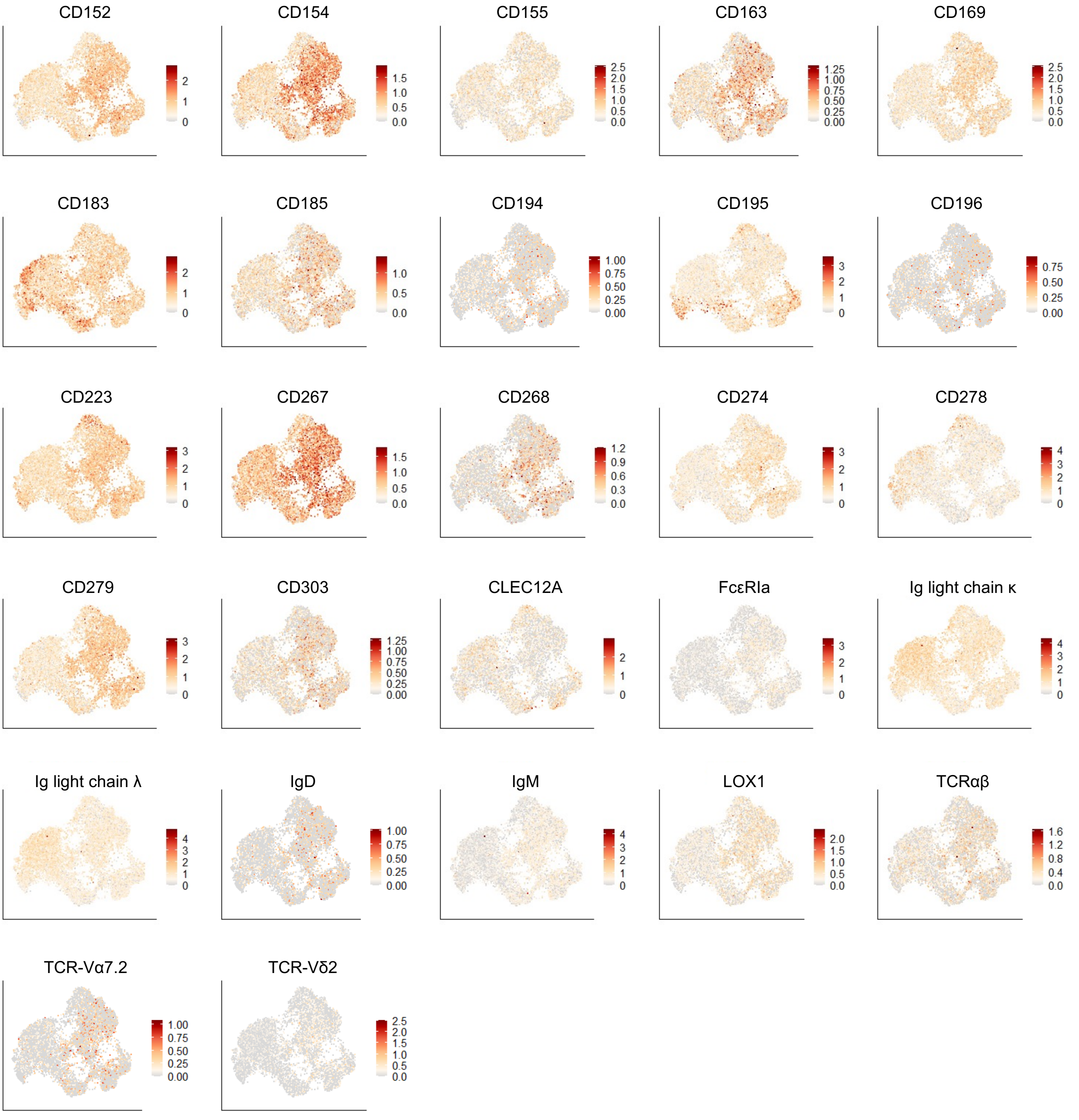
CD152
CD154
CD155
CD163
CD169
CD183
CD185
CD194
CD195
CD196
CD223
CD267
CD268
CD274
CD278
CD279
CD303
CLEC12A
FcεRIa
Ig light chain κ
Ig light chain λ
IgD
IgM
LOX1
TCRαβ
TCR-Vα7.2
TCR-Vδ2

Supplementary Figure 5. WNN-UMAPs depicting each of the 130 surface proteins detected with CITE-seq oligo-labelled mAbs. WNN-UMAPs are separated by proteins commonly associated with (A) NK developmental stages, (B) adhesion, (C) activation, (D) inhibition, (E) cell death, (F) migration, and (G) remaining markers.

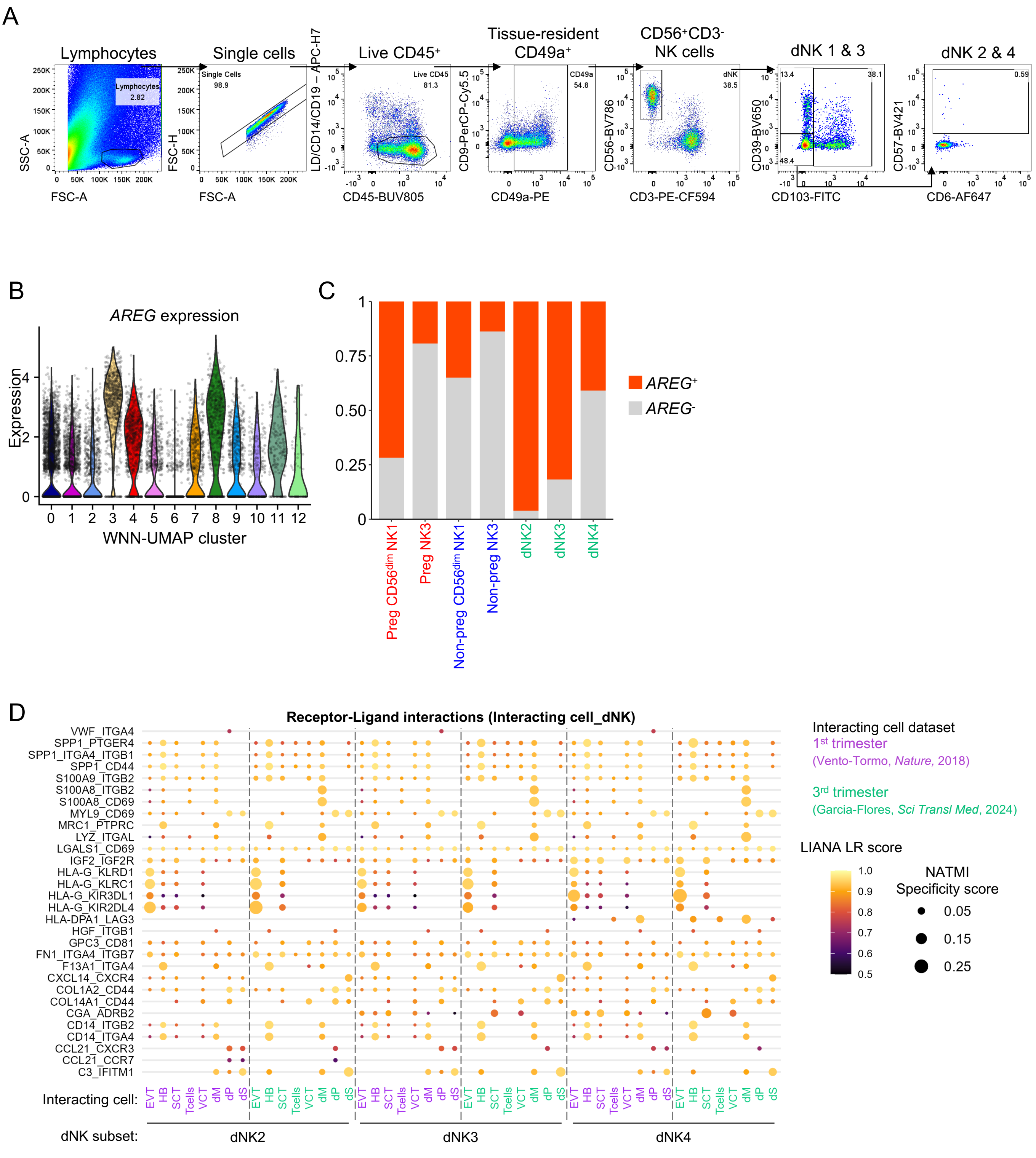

A
Lymphocytes
Single cells
Live CD45⁺
Tissue-resident CD49a⁺
CD56⁺CD3⁻ NK cells
dNK 1 & 3
dNK 2 & 4
SSC-A
FSC-A
FSC-H
LD/CD14/CD19 – APC-H7
CD45-BUV805
CD9-PerCP-Cy5.5
CD49a-PE
CD56-BV786
CD3-PE-CF594
CD39-BV650
CD103-FITC
CD57-BV421
CD6-AF647
B
AREG expression
Expression
WNN-UMAP cluster
C
AREG⁺
AREG⁻
Preg CD56dim NK1
Preg NK3
Non-preg CD56dim NK1
Non-preg NK3
dNK2
dNK3
dNK4
D
Receptor-Ligand interactions (Interacting cell_dNK)
Interacting cell dataset
1st trimester
(Vento-Tormo, Nature, 2018)
3rd trimester
(Garcia-Flores, Sci Transl Med, 2024)
LIANA LR score
NATMI Specificity score
Interacting cell:
dNK subset:
dNK2
dNK3
dNK4

Supplementary Figure 6. dNK subsets and receptor-ligand interactions. (A) Flow cytometry gating strategy from a representative sample to analyse tissue-resident $CD49a^{+}$ $CD56^{+}CD3^{-}$ dNK cells, followed by separation into dNK subsets based on CD39, CD103, and CD57. (B) Violin plot depicting *AREG* expression across WNN-UMAP clusters. (C) Proportion plot showing the frequencies of *AREG*$^{+}$ and *AREG*$^{-}$ NK cells across pregnant and non-pregnant peripheral blood classical $CD56^{dim}$ NK1 cells and adaptive-like $CD56^{bright}$ NK3 cells, and dNK2/3 and adaptive-like dNK4 cells. (D) DotPlot depicting top 20 receptor-ligand interactions based on aggregate rank between dNK2, 3 and 4 subsets described in this study, and decidual cells from first trimester (Vento-Tormo dataset) and third trimester (Garcia-Flores dataset). Receptor-ligand interactions are written as “interacting-cell_dNK-cell”. LIANA LR score reflects the strength of the interaction, and NATMI score reflects the specificity of the interaction.

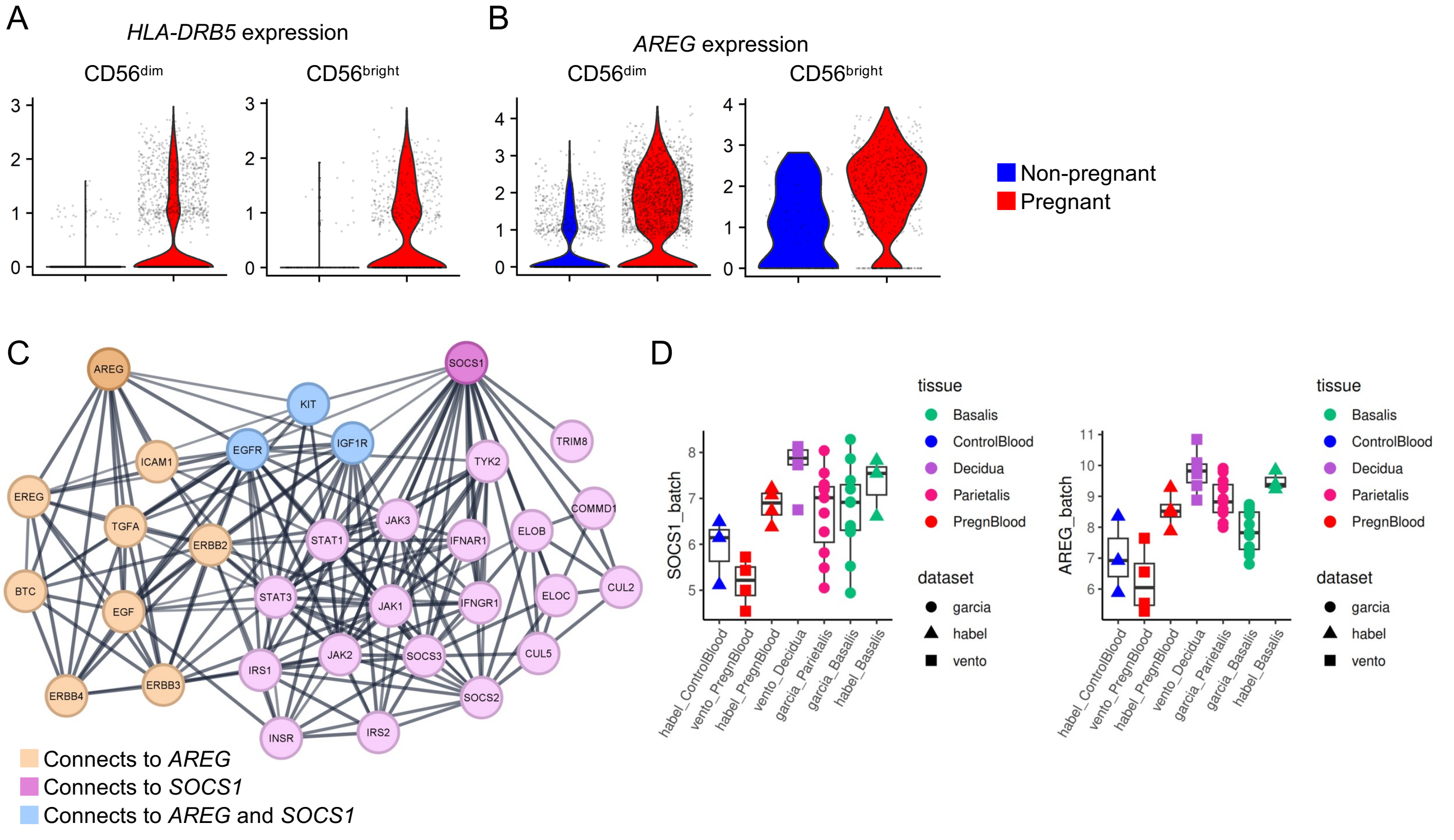
A
HLA-DRB5 expression
CD56dim
CD56bright
B
AREG expression
CD56dim
CD56bright
Non-pregnant
Pregnant
C
AREG
SOCS1
KIT
EGFR
IGF1R
TRIM8
ICAM1
TYK2
EREG
TGFA
JAK3
COMMD1
ERBB2
STAT1
IFNAR1
ELOB
BTC
EGF
STAT3
JAK1
IFNGR1
ELOC
CUL2
JAK2
SOCS3
CUL5
IRS1
ERBB4
ERBB3
SOCS2
INSR
IRS2
Connects to AREG
Connects to SOCS1
Connects to AREG and SOCS1
D
SOCS1_batch
AREG_batch
tissue
Basalis
ControlBlood
Decidua
Parietalis
PregnBlood
dataset
garcia
habel
vento
habel_ControlBlood
vento_PregnBlood
habel_PregnBlood
vento_Decidua
garcia_Parietalis
garcia_Basalis
habel_Basalis

Supplementary Figure 7. HLA-D, *AREG* and *SOCS1* expression across subsets and datasets. (A-B) Violin plot of (A) *HLA-DRB5* and (B) *AREG* expression in CD56^{dim}/NK1 and CD56^{bright}/NK3 cells from pregnant and non-pregnant peripheral blood. (C) Protein-protein interaction network showing the top 30 interactions for a query of *AREG* and *SOCS1* in the STRING database. (D) Pseudobulk expression levels of *SOCS1* and *AREG* in non-pregnant (n=3; Habel dataset), first trimester pregnant blood (n=4; Vento-Tormo dataset), third trimester pregnant blood (n=3; this study's dataset (Habel)), first trimester decidua (n=5; Vento-Tormo dataset), third trimester decidua parietalis (n=10; Garcia-Flores dataset), third trimester decidua basalis (n=9; Garcia-Flores dataset), and third trimester decidua basalis (n=3; Habel dataset).

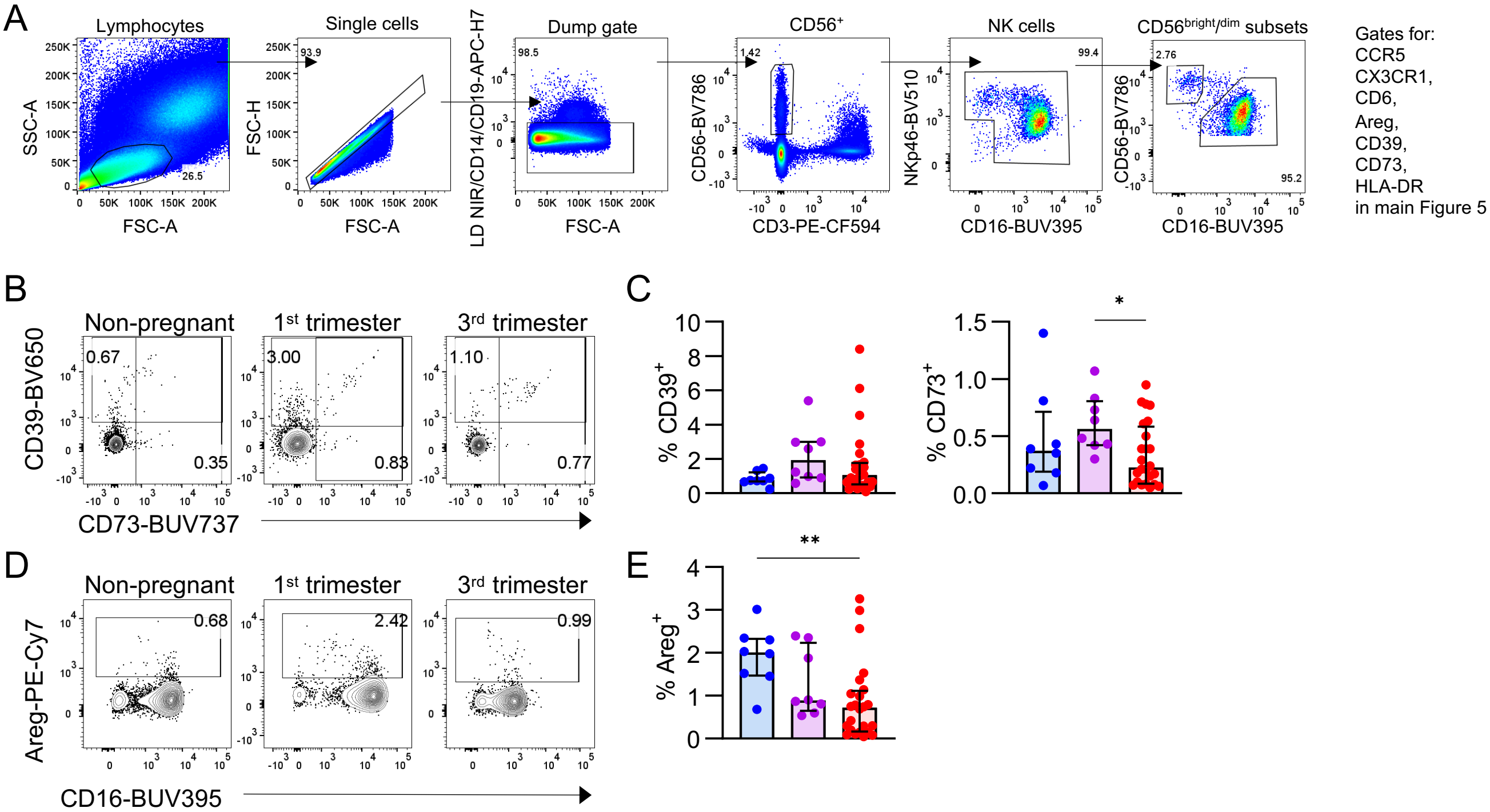
A
Lymphocytes
Single cells
Dump gate
CD56+
NK cells
CD56bright/dim subsets
SSC-A
FSC-A
FSC-H
LD NIR/CD14/CD19-APC-H7
CD56-BV786
CD3-PE-CF594
NKp46-BV510
CD16-BUV395
26.5
93.9
98.5
1.42
99.4
2.76
95.2
Gates for:
CCR5
CX3CR1,
CD6,
Areg,
CD39,
CD73,
HLA-DR
in main Figure 5
B
Non-pregnant
1st trimester
3rd trimester
CD39-BV650
CD73-BUV737
0.67
0.35
3.00
0.83
1.10
0.77
C
% CD39+
% CD73+
*
D
Areg-PE-Cy7
CD16-BUV395
0.68
2.42
0.99
E
% Areg+
**

Supplementary Figure 8. Whole blood staining to validate differentially expressed markers in circulating NK cells. (A) Flow cytometry gating strategy from one representative whole blood staining for NK cell panel outlined in Supplementary Tables 3-4. (B-C) Representative FACS plots (B) and frequencies of $CD39^+$ and $CD73^+$ (C) NK cells in non-pregnant (n=8), first trimester pregnant (n=8), and third trimester pregnant (n=24). (D-E) Representative FACS plots (D) and frequencies of $Areg^+$ (E) NK cells in non-pregnant (n=8), first trimester pregnant (n=8), and third trimester pregnant (n=24).
