# Supplementary material for: Multidimensional molecular profiling reveals decidual and circulating NK cell dynamics in early and late pregnancy
Source: eBioMedicine. 2026 Oct 1;132:106495. doi: 10.1016/j.ebiom.2026.106495 (PMC13657211; doi:10.1016/j.ebiom.2026.106495)
Supplement: Supplementary file 2 [file mmc18.pdf]

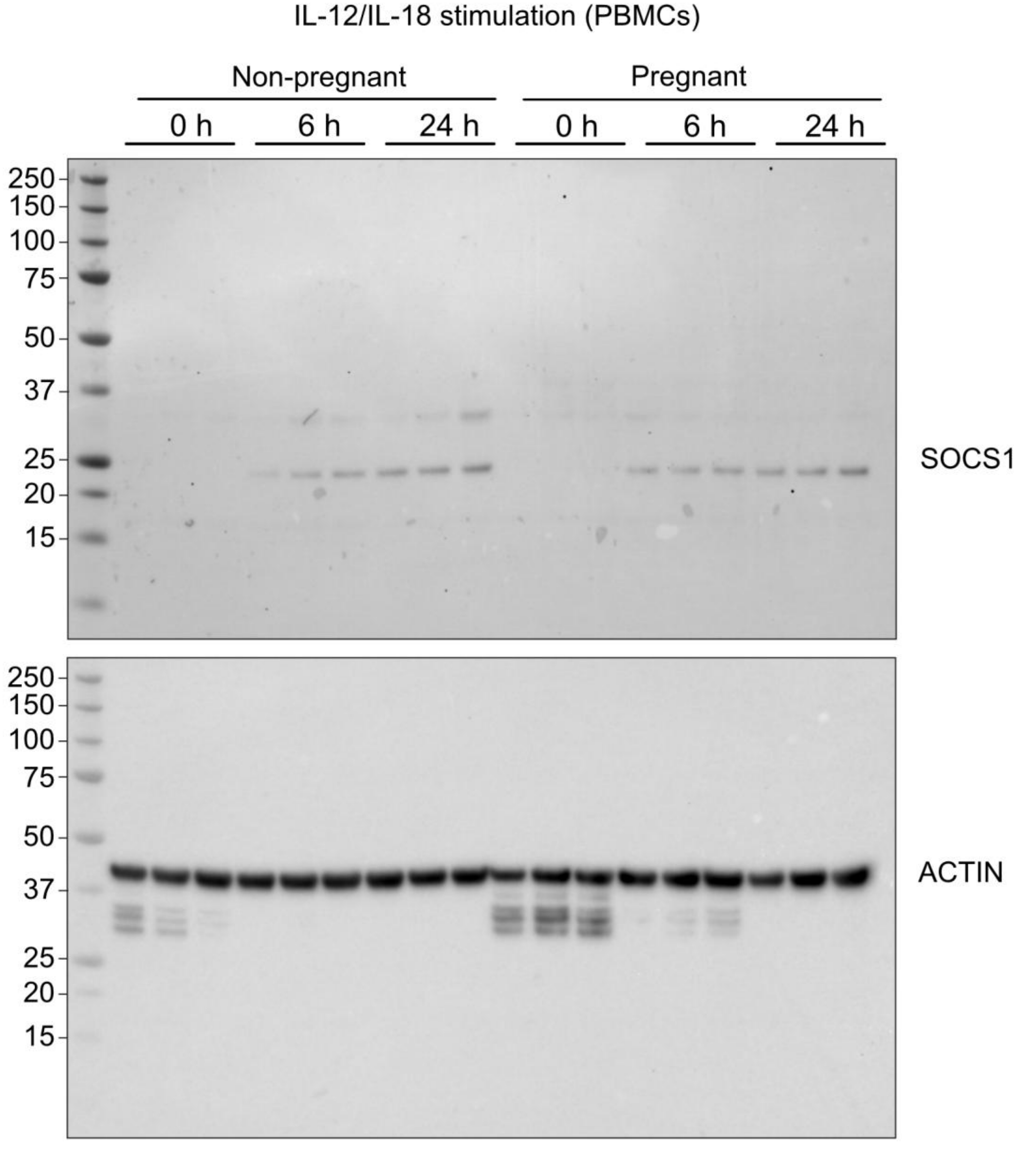
IL-12/IL-18 stimulation (PBMCs)
Non-pregnant
Pregnant
0 h
6 h
24 h
0 h
6 h
24 h
250
150
100
75
50
37
25
20
15
SOCS1
250
150
100
75
50
37
25
20
15
ACTIN

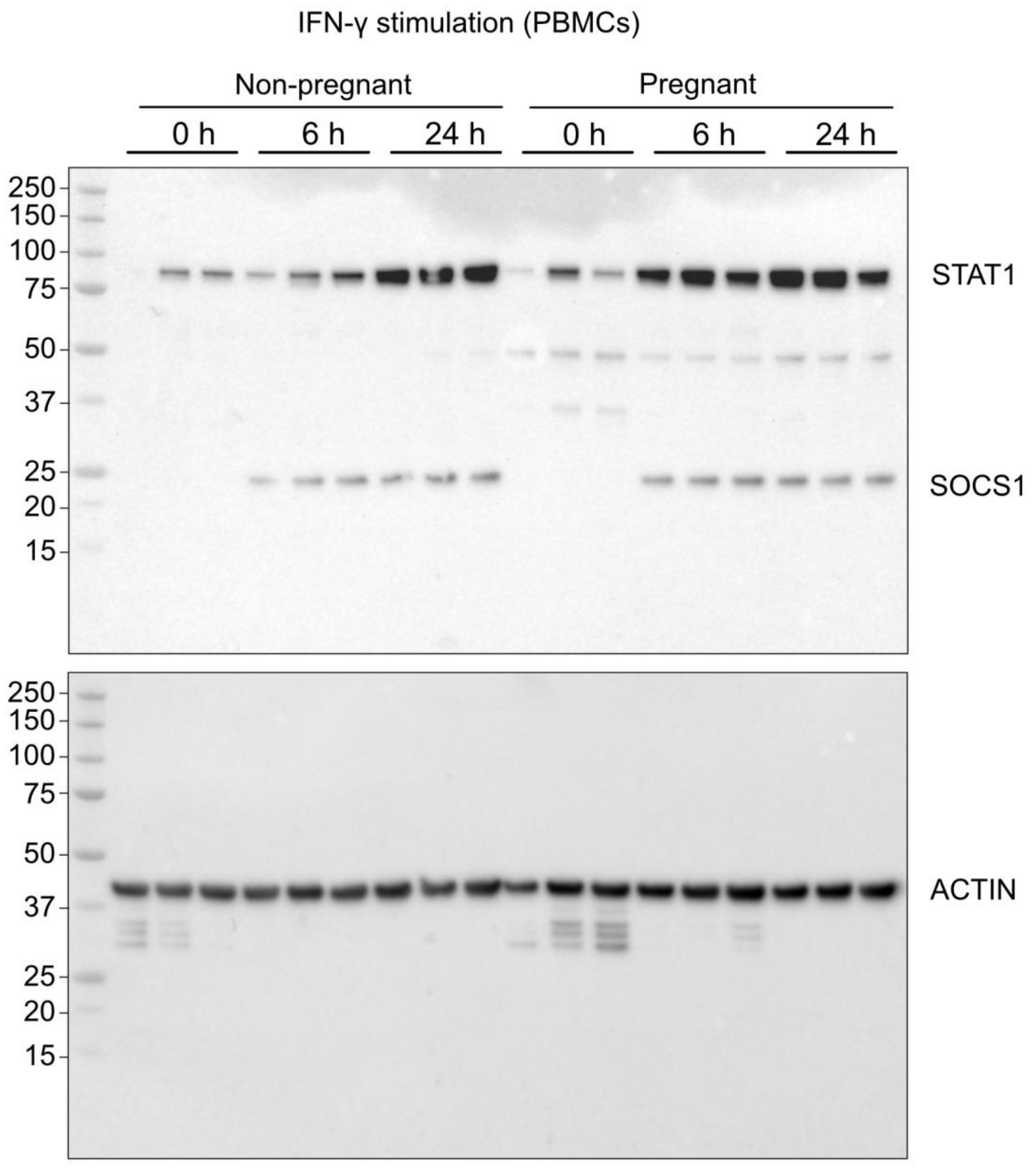
IFN-γ stimulation (PBMCs)
Non-pregnant
Pregnant
0 h
6 h
24 h
0 h
6 h
24 h
250
150
100
75
50
37
25
20
15
STAT1
SOCS1
ACTIN
